# Lgmn targets two distinct GPCRs, PAR2 and µ-OR1, and induces cell death in acute lymphoblastic leukemia through an intracellular Ca²⁺ imbalance triggered by ER Ca²⁺ release

**DOI:** 10.1038/s41420-026-03003-3

**Published:** 2026-03-07

**Authors:** Jung Kwon Lee, Karl Riabowol, Ki-Young Lee

**Affiliations:** 1 Department of Biochemistry & Molecular Biology, Arnie Charbonneau Cancer and Alberta Children’s Hospital Research Institutes, University of Calgary, Calgary, AB Canada; 2 Department of Cell Biology & Anatomy, Arnie Charbonneau Cancer and Alberta Children’s Hospital Research Institutes, University of Calgary, Calgary, AB Canada; 3 Department of Biochemistry & Molecular Biology and Oncology, Arnie Charbonneau Cancer and Alberta Children’s Hospital Research Institutes, University of Calgary, Calgary, AB Canada

**Keywords:** Acute lymphocytic leukaemia, Cancer prevention

## Abstract

Legumain (Lgmn) is a virulence factor found in the protozoan parasites *Blastocystis* and *Trichomonas*, which affect both humans and animals. However, its specific targets and cytotoxic mechanisms on host cells are not well understood. Recent findings show that Lgmn cleaves PAR2 at the N_30_-R_31_ residue, a site also targeted by L-asparaginase, a vital treatment for acute lymphoblastic leukemia (ALL), a severe hematologic cancer that poses high risks to children. This emphasizes the urgent need for more effective therapeutic strategies. Here, we demonstrate that Lgmn induces ER Ca^2+^ release via the µ-OR1-G_αi_ and PAR2-G_αq_ pathways. In PAR2-knockdown ALL cells, the stimulation of adenylate cyclase (AC) with forskolin or treatment with 8-CPT-cAMP effectively inhibits Lgmn-induced µ-OR1-mediated ER Ca^2+^ release, indicating that Lgmn’s stimulation of µ-OR1 results in the downregulation of AC and a subsequent decrease in cAMP levels. Additionally, the PKA-specific inhibitor 14–22 amide alone triggers ER Ca^2+^ release, and subsequent treatment with Lgmn does not enhance this effect, suggesting that PKA inhibition plays a role and that the Lgmn-µ-OR1-AC-cAMP axis can be bypassed in µ-OR1-mediated ER Ca^2+^ release. Furthermore, we observed a corresponding reduction in the phosphorylation of PLCβ3 at Ser1105 and BAD at Ser118, both of which are regulated by PKA. The Lgmn-induced ER Ca^2+^ release ultimately leads to apoptosis in ALL cells, which can be reversed by blocking ER Ca^2+^ release. Our results thus provide novel insights into the specific targets of Lgmn secreted from the protozoa and demonstrate how this virulence factor induces cytotoxic effects on host cells, paving the way for innovative therapeutic strategies for patients with ALL.

## Introduction

Legumain (Lgmn; EC 3.4.22.34) is an enzyme that hydrolyzes Asn/Asp–Xaa bonds found in peptides and proteins [1–3], and it is classified as a member of the C13 family of cysteine proteases [4, 5]. This enzyme is present in the culture medium of protozoa [6], such as *Blastocystis* and *Trichomonas*. Lgmn plays a vital role in the virulence and pathogenicity of these parasites, while also modulating host immune responses. It facilitates the invasion of epithelial cells and can induce apoptosis. However, the specific targets of Lgmn and the exact molecular mechanisms through which it exerts its cytotoxic effects on host cells remain obscure.

Recent research on the G-protein-coupled receptor (GPCR) known as protease-activated receptor 2 (PAR2), which is associated with pain in oral squamous cell carcinoma (OSCC) [7], has shed light on identifying the targets of Lgmn related to its mechanism of action. The study revealed that Lgmn can mobilize intracellular Ca²⁺ in HEK293 human embryonic kidney cells by specifically targeting PAR2, particularly its N-terminal residues, N_30_-R_31_ [7]. However, the study also showed that Lgmn continues to induce Ca²⁺ mobilization in both PAR2-knockdown HEK293 cells and those expressing a PAR2 N30A substituted variant (PAR2KO/N30A), which is resistant to Lgmn cleavage [7]. This implies that Lgmn may trigger the mobilization of intracellular Ca^2+^ through an alternative upstream regulator that does not involve PAR2. However, the identity of this alternative target and the molecular mechanisms by which Lgmn causes intracellular Ca²⁺ mobilization still need to be elucidated.

Like Lgmn, L-asparaginase also displays PAR2 protease activity [8]. This enzyme cleaves the N-terminal extracellular domain of PAR2 at the N_30_-R_31_ and R_31_-S_32_ residues [8], leading to IP3R-mediated release of Ca^2+^ from the endoplasmic reticulum (ER). This results in a significant rise in intracellular calcium concentration [Ca^2+^]_i_, triggering apoptosis in acute lymphoblastic leukemia (ALL) cells through the activation of the Ca^2+^-regulated caspase pathway [9]. Additionally, L-asparaginase targets another GPCR, µ-OR1, contributing to these effects [10]. Thus, L-asparaginase has become a fundamental therapeutic option for individuals diagnosed with ALL, a very aggressive blood cancer that primarily impacts children. Although improvements in aggressive chemotherapy have enhanced long-term survival rates, major challenges related to relapse and side effects still remain [11]. Thus, efforts are ongoing to develop more effective therapeutic strategies.

The GPCR, PAR2, primarily interacts with G_αq_, leading to the direct activation of PLCβ3 [10], which cleaves PIP2 into diacylglycerol (DAG) and IP3. In contrast, the activation of µ-OR1 results in a conformational alteration, facilitating GTP exchange on the G_α_ subunit. This separates G_α_ from G_βγ_ [12], instigating signaling through downstream effectors: i.e., Ca^2+^ and cAMP. The G_βγ_ dimer activates PLCβ, resulting in IP3-mediated release of Ca^2+^ from the ER. Simultaneously, GTP-bound G_αi_ inhibits adenylyl cyclase (AC), lowering cAMP levels [13]. This decrease in cAMP reduces PKA activity, promoting PLCβ3 activity [14] and IP3R-mediated Ca^2+^ release, while also facilitating the BCL2-BAD interaction, diminishing BCL2’s anti-apoptotic effect [15, 16]. This ultimately allows BAK and BAX to form pores in the mitochondrial membrane, triggering apoptosis through cytochrome C release and caspase activation [17, 18]. Whether Lgmn targets these two distinct GPCRs to mobilize intracellular Ca^2+^ and how it engages its downstream effectors remains an area for further exploration.

In this study, we employed knockdown strategies, site-directed mutagenesis, single-cell Ca^2+^ imaging, and small molecular agonists and antagonists to identify µ-OR1 and PAR2 as novel targets of Lgmn and elucidate the molecular mechanism by which Lgmn causes apoptosis in ALL cells. We demonstrated that Lgmn specifically targets two distinct GPCRs, PAR2 and µ-OR1, leading to the release of Ca^2+^ from the ER across ALL cell subtypes. Notably, µ-OR1 plays a predominant role, whereas PAR2 has a comparatively minor impact on Lgmn-induced ER Ca²⁺ release. Utilizing PAR2- and µ-OR1-knockdown ALL cells, alongside small molecular agonists and antagonists, we show that Lgmn-mediated ER Ca²⁺ release operates through the µ-OR1-G_αi_-AC-↓[cAMP]_i_-↓PKA-↓pSer1105-PLCβ3 and PAR2-G_αq_-PLCβ3 signaling pathways, ultimately resulting in apoptosis in ALL cells. Importantly, this process can be reversed by blocking the release of ER Ca^2+^. Thus, our studies identify µ-OR1 and PAR2 as Lgmn targets, and elucidate the molecular mechanism by which Lgmn causes apoptosis in ALL cells.

## Results

### Lgmn targets two discrete GPCRs, PAR2 and µ-OR1, to elicit ER Ca^2+^ release in both HEK293 and ALL cells

**Fig. 1:**
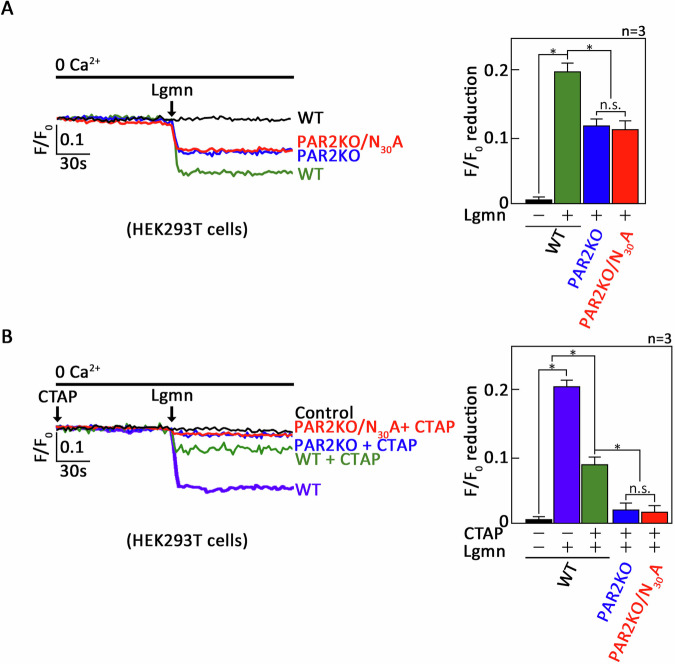
Lgmn stimulates PAR2 and µ-OR1, leading to Ca^2+^ release from the ER in HEK293 cells. Wild-type and PAR2-knockdown HEK293T cells (PAR2KO), along with PAR2-knockdown HEK293T cells expressing a hPAR2 N_30_A substituted form (PAR2KO/N_30_A), preloaded with Mag-Fluo-4 AM, were treated without (**A**) or with (**B**) CTAP, before exposure to Lgmn. Subsequent analyses were conducted using single-cell Ca^2+^ imaging to evaluate ER Ca^2+^ release. The left panels in (**A**, **B**) display average Ca^2+^ traces recorded every 2 s from 20 individual cells, both prior to and following Lgmn treatment. The data presented are from one of three independent experiments (*n* = 3) that produced similar results. The chart on the right illustrates the differences in ER Ca^2+^ release following Lgmn treatment. An *F*/*F*_0_ value measured 30 s after the addition of Lgmn was used to assess the reduction in *F*/*F*_0_ for the right panels of (**A**, **B**). The values are expressed as means ± SEM from three independent experiments, with *p < 0.05 indicating statistical significance; “N.S.” denotes not significant.

Previous studies have shown that Lgmn cleaves the N-terminal N_30_-R_31_ residue of PAR2, leading to PAR2-driven Ca²⁺ mobilization in HEK293 cells [7]. However, earlier studies did not clarify whether this effect was due to Ca²⁺ release from the ER. Thus, we first aimed to determine if Lgmn induces ER Ca²⁺ release in HEK293 cells. To do so, we loaded these cells with the ER Ca²⁺ probe, Mag-Fluo-4 AM [19], and subsequently treated them with Lgmn. The analysis of ER Ca²⁺ release was performed using single-cell Ca²⁺ imaging. As illustrated in Fig. 1A, Lgmn elicited a significant release of Ca²⁺ from the ER in HEK293 cells. Next, we sought to investigate whether the observed ER Ca²⁺ release is solely due to Lgmn’s stimulation of PAR2, specifically targeting the N-terminal N_30_-R_31_ residue [7]. For this purpose, we utilized PAR2-knockdown HEK293 cells (PAR2KO) that expresses a PAR2 N_30_A substituted form (PAR2KO/N_30_A), which is resistant to cleavage by Lgmn [7]. Wild-type and PAR2-knockdown HEK293 cells served as positive and negative controls, respectively. Interestingly, we found that both the PAR2KO and PAR2KO/N_30_A still induced substantial ER Ca²⁺ release, although a reduction in ER Ca²⁺ release was observed in these cells (Fig. 1A). This finding suggests that Lgmn triggers ER Ca^2+^ release through an alternative upstream regulator, one that is not PAR2. Previous studies have demonstrated that a GPCR, µ-OR1, also stimulates Ca^2+^ release from the ER [9, 10, 20]. To investigate this possibility, we pretreated both PAR2KO and PAR2KO/N_30_A cells with CTAP [21], a potent inhibitor of µ-OR1, before exposing them to Lgmn and analyzing through single-cell Ca²⁺ imaging. As depicted in Fig. 1B, HEK293 cells pretreated with CTAP exhibited a significant reduction in ER Ca²⁺ release compared to untreated control HEK cells (Fig. 1B). Moreover, ER Ca²⁺ release was completely abolished in both PAR2KO and PAR2KO/N_30_A cells that had been pre-treated with CTAP. These findings indicate that Lgmn targets two distinct GPCRs, PAR2 and µ-OR1, to induce ER Ca^2+^ release in HEK293 cells. Furthermore, the markedly reduced level of Lgmn-induced ER Ca^2+^ release in HEK293 cells pretreated with CTAP highlights the significant role of µ-OR1 in mediating this process.

**Fig. 2:**
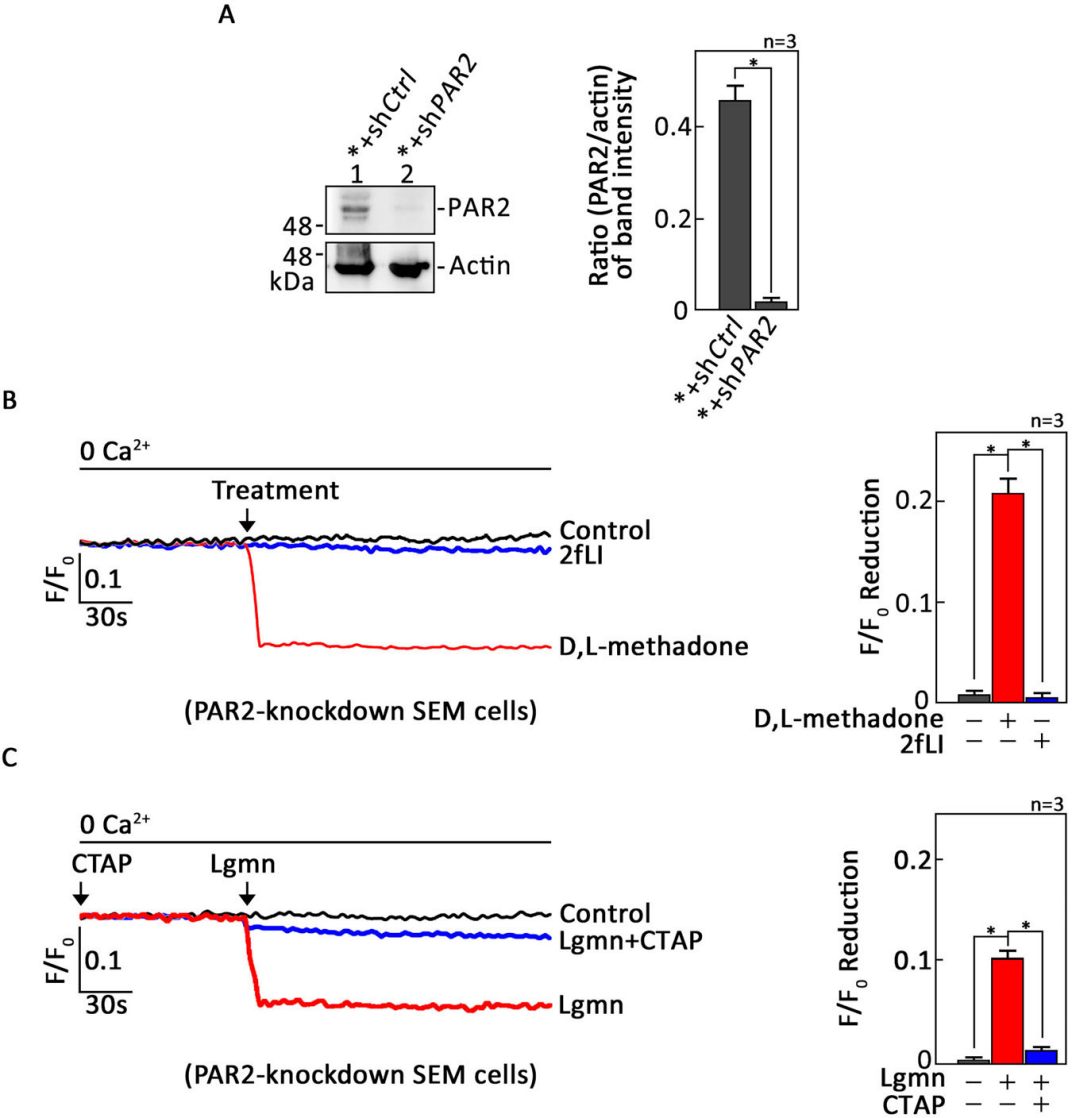
Pretreatment of PAR2-knockdown SEM cells with CTAP resulted in the elimination of ER Ca^2+^ release, indicating that Lgmn targets both PAR2 and µ-OR1. **A** Lysates from SEM B-ALL cells (*) infected with lentivirus carrying control (*+sh*Ctrl*) or PAR2 shRNA (*+sh*PAR2*) were analyzed using SDS-PAGE and subsequently immunoblotted for PAR2. An actin blot was used as a loading control. The panel on the right presents the ratios of PAR2 to actin levels, as determined by densitometric analysis of the blots via NIH ImageJ 1.61. Actin levels were normalized to 1.0, and standard deviations were calculated based on three independent experiments (*n* = 3). *+sh*PAR2* cells loaded with Mag-Fluo-4 AM were pretreated without (**B**) or with (**C**) CTAP, prior to exposure to a PAR2 agonist, 2-furoyl-LIGRLO-NH_2_ (2fLI; **B**), or D,L-methadone (**B**), or Lgmn (**C**). Single-cell Ca^2+^ imaging was performed to measure ER Ca^2+^ release. The left panels in (**B**, **C**) display average Ca^2+^ traces recorded every 2 s from 20 individual cells, before and after treatment with 2fLI, D,L-methadone, or Lgmn. The data presented are from one of three independent experiments (*n* = 3) that yielded similar results. The chart on the right illustrates the differences in ER Ca^2+^ release following treatment with 2fLI, D,L-methadone, or Lgmn. An *F*/*F*_0_ value measured 30 s after the addition of 2fLI, D,L-methadone, or Lgmn was used to assess *F*/*F*_0_ reduction in the right panels of (**B**, **C**). The values are expressed as means ± SEM from three independent experiments, with *p < 0.05 indicating statistical significance.

We then examined whether the findings observed in HEK293 cells are also applicable to acute lymphoblastic leukemia (ALL) cells. For this experiment, we utilized SEM cells (*) derived from the peripheral blood of a 5-year-old female patient with relapsed B-ALL [8–10, 22, 23]. These cells were loaded with Mag-Fluo-4 AM and pretreated with CTAP or GB83, a potent PAR2 antagonist [24], before being exposed to either a synthetic opioid, D,L-methadone, or a synthetic PAR2 agonist peptide, 2-furosyl-LIGRLO-NH_2_ (2fLI), respectively. As expected, the pretreatment with CTAP or GB83 completely inhibited D,L-methadone (Supplementary Fig. 1A) and 2fLI (Supplementary Fig. 1B)-induced ER Ca²⁺ release in SEM cells, respectively, confirming the specific inhibition of µ-OR1 and PAR2-mediated ER Ca²⁺ release by CTAP and GB83. Next, the SEM cells (*) were infected with lentivirus carrying sh*PAR2* (*+sh*PAR2*) to achieve *PAR2* knockdown (Fig. 2A: see uncropped blots in Supplemental Material). They were subsequently loaded with Mag-Fluo-4 AM and exposed to 2fLI. Our single-cell Ca^2+^ imaging revealed that *+sh*PAR2* cells were unable to induce PAR2-mediated ER Ca^2+^ release (Fig. 2B), confirming the successful knockdown of PAR2 in these cells. However, treatment with D.L-methadone did trigger ER Ca^2+^ release in these cells, indicating the presence of an active µ-OR1-mediated signaling pathway. We then evaluated the effect of Lgmn on ER Ca^2+^ release in the *+sh*PAR2* cells loaded with Mag-Fluo-4 AM. Treatment with Lgmn resulted in ER Ca^2+^ release (Fig. 2C), suggesting that, similar to HEK293 cells, Lgmn induces ER Ca^2+^ release through an alternative upstream regulator distinct from PAR2. Pre-treatment of Mag-Fluo-4 AM-loaded *+sh*PAR2* cells with CTAP drastically reduced the level of ER Ca^2+^ release (Fig. 2C, right panel, bars 2 vs 3) following Lgmn treatment, underscoring the important role of µ-OR1 in mediating this release from the ER.

**Fig. 3:**
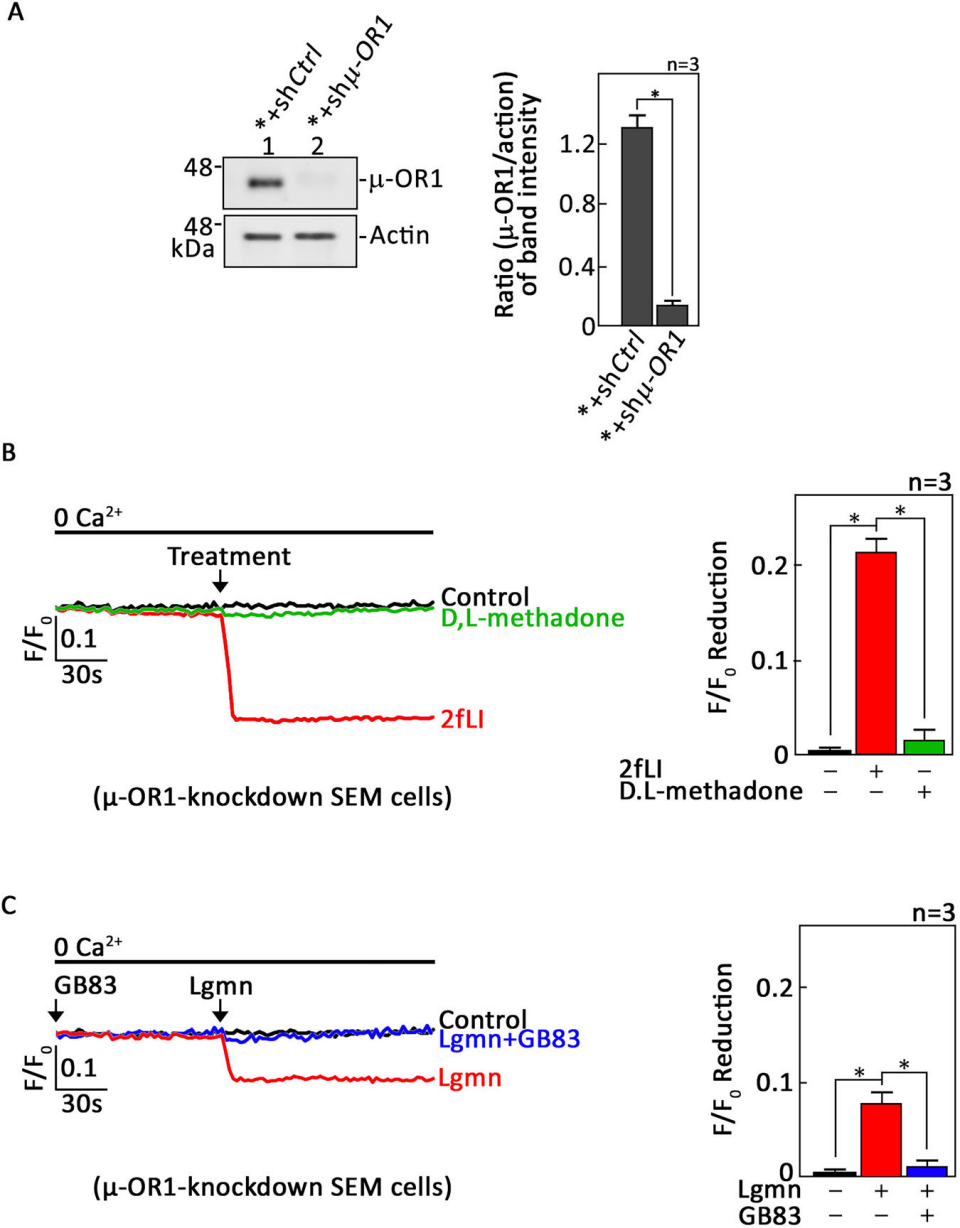
Pretreatment of µ-OR1-knockdown SEM cells with GB83 resulted in the complete inhibition of ER Ca^2+^ release, demonstrating that Lgmn targets both PAR2 and µ-OR1. **A** Lysates from SEM cells (*) infected with lentivirus carrying either control (*+sh*Ctrl*) or µ-OR1 shRNA (*+sh*µ-OR1*) underwent SDS-PAGE and were subsequently immunoblotted for µ-OR1. Actin blot served as loading controls. The right panel displays the ratios of µ-OR1 to actin levels, determined through densitometric analysis of the blots using NIH ImageJ 1.61, with actin levels normalized to 1.0. Standard deviations were calculated from three experimental sets (*n* = 3). *+sh*µ-OR1* cells, loaded with Mag-Fluo-4 AM, were pretreated without (**B**) or with (**C**) GB83, before being exposed to 2fLI, D,L-methadone, or Lgmn. Single-cell Ca^2+^ imaging was performed to evaluate ER Ca^2+^ release. The left panels in (**B**, **C**) display average Ca^2+^ traces recorded every 2 s from 20 individual cells, both prior to and following treatment with 2fLI, D,L-methadone, or Lgmn. The data illustrated are from one of three independent experiments (*n* = 3) that produced consistent results. The chart on the right highlights the differences in ER Ca^2+^ release following treatment with 2fLI, D,L-methadone, or Lgmn. An *F*/*F*_0_ value measured 30 s after the addition of 2fLI, D,L-methadone, or Lgmn was used to evaluate *F*/*F*_0_ reduction for the right panels of (**B**, **C**). The values are expressed as means ± SEM from the three independent experiments, with *p < 0.05 indicating statistical significance.

We also used SEM cells (*) infected with lentivirus carrying sh*µ-OR1* (*+sh*µ-OR1*) to knock down *µ-OR1* (Fig. 3A: see uncropped blots in Supplemental Material). The cells were then loaded with Mag-Fluo-4 AM and exposed to either D,L-methadone or 2fLI. Single-cell Ca^2+^ imaging confirmed that *+sh*µ-OR1* cells could not induce µ-OR1-mediated ER Ca^2+^ release (Fig. 3B), validating the effective loss of µ-OR1 in these cells. Conversely, 2fLI evoked ER Ca^2+^ release in these cells, serving as a positive control. Consistent with our findings in *+sh*PAR2* cells, Lgmn also induced ER Ca^2+^ release in *+sh*µ-OR1* cells, indicating that PAR2 facilitates this response (Fig. 2C). Notably, Lgmn-induced ER Ca^2+^ release was completely inhibited in Mag-Fluo-4 AM-loaded *+shµ-OR1 cells that had been pre-treated with GB83 (Fig. 2C, right panel, bars 2 vs 3). This suggests that Lgmn targets two distinct GPCRs, PAR2 and µ-OR1, to elicit ER Ca^2+^ release in ALL cells.

### µ-OR1 plays a major role in Lgmn-induced ER Ca²^+^ release, while PAR2 has a comparatively minor role across ALL cell types

**Fig. 4:**
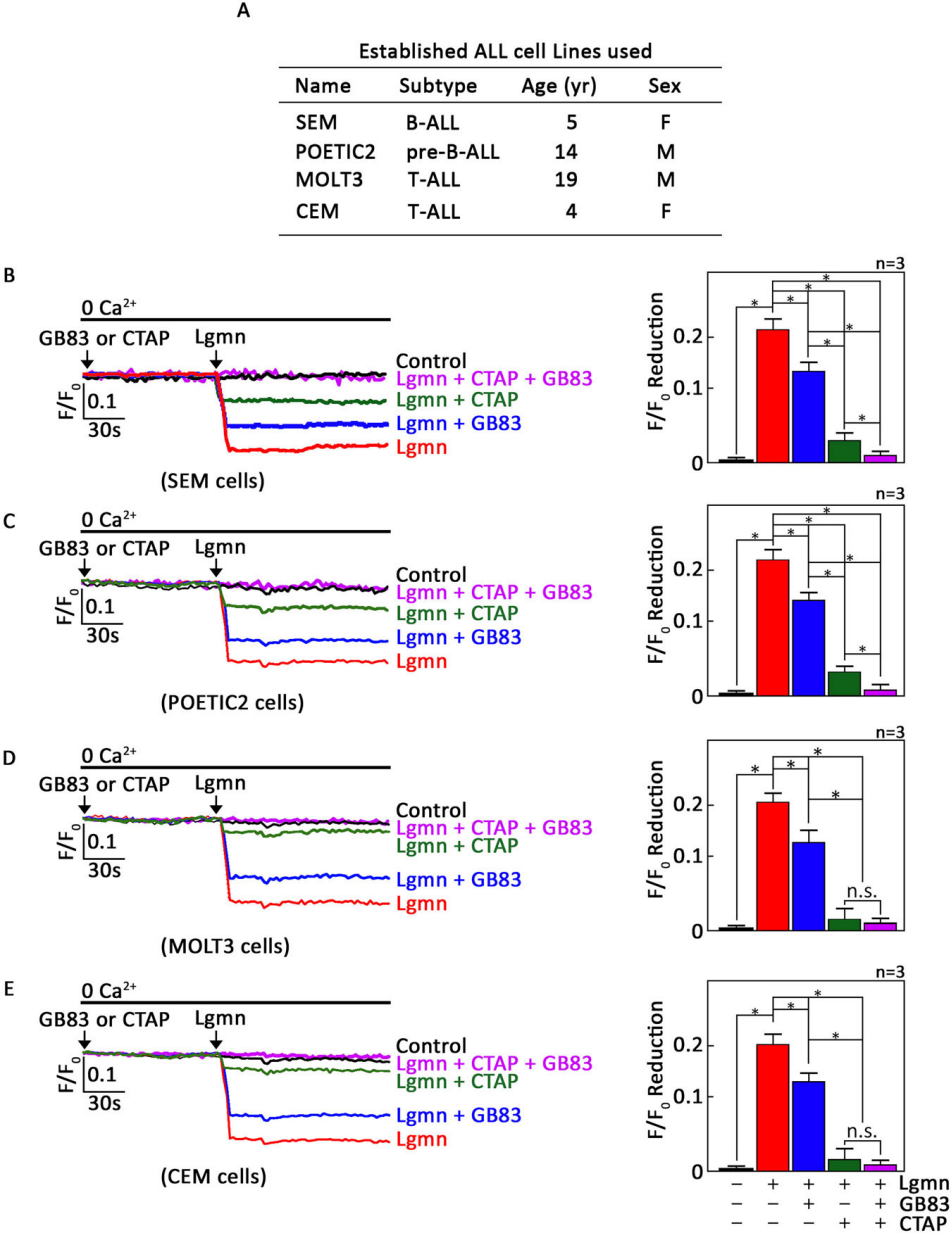
illustrates that Lgmn-induced ER Ca²^+^ release is primarily mediated by µ-OR1, with a lesser contribution from PAR2 across ALL cell types examined. **A** An overview of the characteristics of the established B- and T-ALL cells utilized in this study. B-ALL [SEM (**B**) & POETIC2 (**C**)] and T-ALL [MOLT3 (**D**) & CEM (**E**)] cells preloaded with Mag-Fluo-4 AM were pretreated (or not) with either CTAP or GB83 or a combination of both. Following treatment, the cells were exposed to Lgmn, and single-cell Ca^2+^ imaging was conducted to assess ER Ca^2+^ release. The left panels in **B**–**E** display the average Ca^2+^ traces recorded every 2 s from 20 individual cells, both before and after Lgmn treatment. The data shown represent one of three independent experiments (*n* = 3), yielding similar results. The chart on the right depicts the variations in ER Ca^2+^ release following Lgmn exposure. An *F*/*F*_0_ value measured 30 s after the addition of Lgmn was used to assess *F*/*F*_0_ reduction for the right panels of (**B**–**E**). The values are expressed as means ± SEM from three independent experiments, with *p < 0.05 indicating statistical significance.

Our observation that the F/F_0_ value in PAR2-knockdown cells following Lgmn treatment is greater (Fig. 2C, right panel, bar 2) than that in µ-OR1-knockdown cells (Fig. 3C, right panel, bar 2) prompted us to further investigate the contributions of PAR2 and µ-OR1 in mediating Lgmn-induced ER Ca²⁺ release. To this end, two representative B-ALL (SEM [8–10, 22, 23] & POETIC2 [10, 20, 23, 25, 26]) and T-ALL (MOLT3 [23] & CEM [27]) cells (Fig. 4A) were loaded with Mag-Fluo-4 AM and pretreated with either CTAP or GB83 or both. These cells were then exposed to Lgmn and analyzed using single-cell Ca²⁺ imaging to assess ER Ca²⁺ release. As shown in Fig. 4B (SEM), 4 C (POETIC2), 4D (MOLT3) and 4 F (CEM), pretreatment with CTAP and GB83 resulted in a reduction of ER Ca²⁺ release following exposure to Lgmn. Notably, CTAP caused a more considerable decrease in ER Ca²⁺ release [74.1 ± 3.1% (SEM), 73.2 ± 2.3% (POETIC2), 69.75 ± 2.8% (MOLT3) & 70.96 ± 3.2% (CEM); right panels, bars 2 vs 4] compared to GB83 [20.5 ± 2.2% (SEM), 21.5 ± 2.5% (POETIC2), 26.9 ± 2.1% (MOLT3) & 23 ± 7% (CEM); right panels, bars 2 vs 3]. These findings indicate that µ-OR1 plays a significant role in Lgmn-mediated ER Ca²⁺ release, whereas the influence of PAR2 appears to be lesser across B- and T-ALL cell subtypes.

### Lgmn-induced ER Ca^2+^ release in ALL cells signals through µ-OR1-G_αi_ and PAR2-G_αq_

**Fig. 5:**
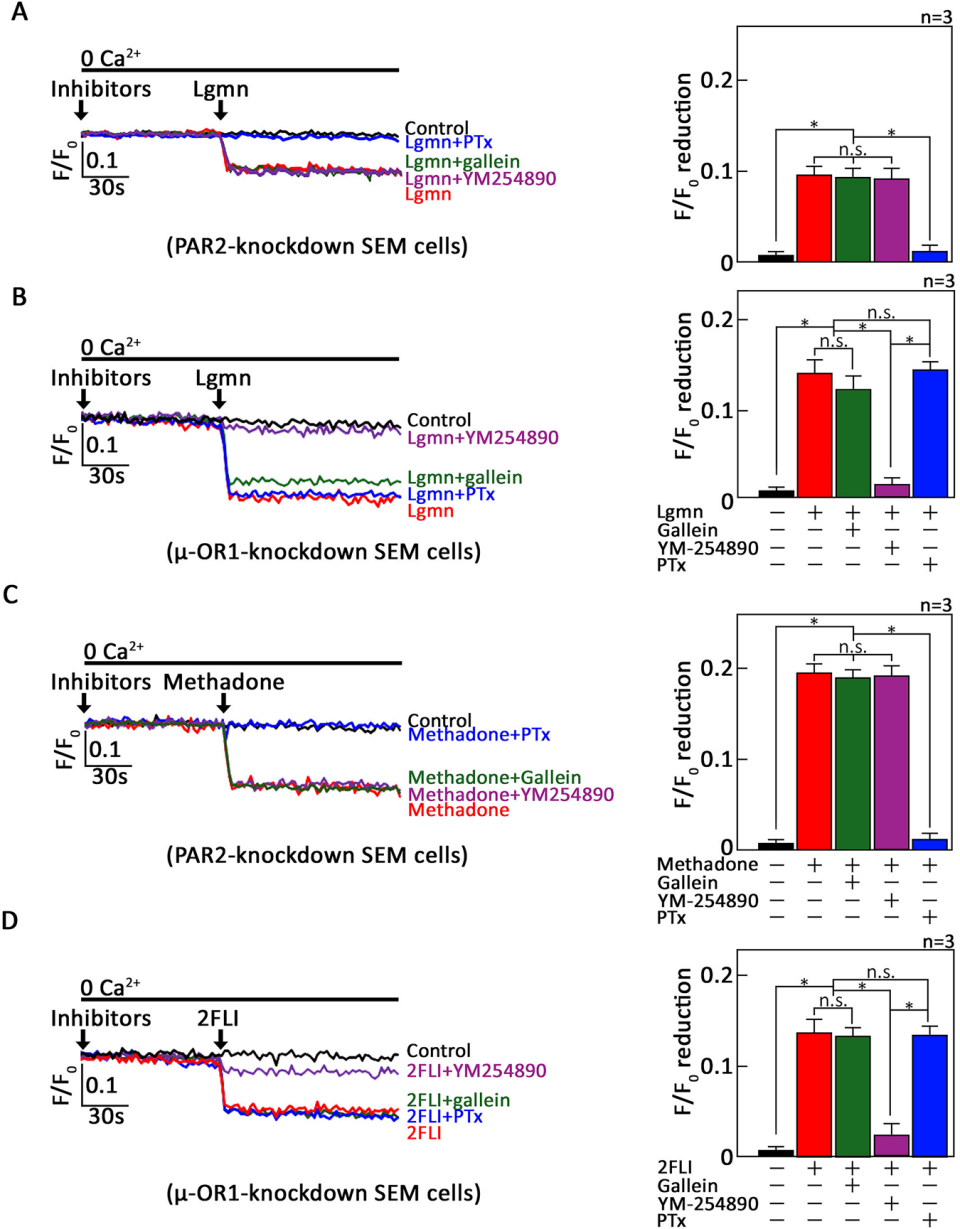
Lgmn induces ER Ca^2+^ release through the µ-OR1-G_αi_ and PAR2-G_αq_ pathways in SEM B-ALL cells. PAR2-knockdown *+sh*PAR2* (**A**, **C**) and µ-OR1-knockdown *+sh*µ-OR1* (**B**, **D**) cells, preloaded with Mag-Fluo-4 AM, were treated (or not) with either PTx or Gallein or YM-254890 prior to exposure to Lgmn (**A**, **B**), D,L-methadone (**C**), and 2fLI (**D**), respectively. Single-cell Ca^2+^ imaging was conducted to evaluate ER Ca^2+^ release. The panels on the left (**A**–**D**) illustrate the average Ca2+ tracing recorded every 2 s from 20 individual cells, both before and after Lgmn treatment. The data presented are from one of three independent experiments (*n* = 3), yielding similar results. The chart on the right depicts the differences in ER Ca^2+^ release after Lgmn treatment. An *F*/*F*_0_ value of 30 s post-addition of Lgmn was utilized to determine F/F_0_ reduction in the right panels of (**A–D**). Values are presented as means ± SEM from three independent experiments, with *p < 0.05 statistical significance; “N.S.” denotes not significant.

Our discovery that Lgmn targets two GPCRs, µ-OR1 and PAR2, to induce ER Ca^2+^ release across ALL cells prompted us to determine the involvement of their downstream G protein mediators in these processes. To achieve this, *+sh*PAR2* and *+sh*µ-OR1* cells loaded with Mag-Fluo-4 AM were pretreated with either a G_αi_ inhibitor PTx [28] or a G_βϒ_ inhibitor Gallein [29] or a G_q_ inhibitor YM-254890 [30], before being exposed to Lgmn, followed by analysis through single-cell Ca²⁺ imaging. As illustrated in Fig. 5A, PTx treatment completely obliterated Lgmn-induced ER Ca^2+^ release in *+sh*PAR2* cells. In contrast, Gallein or YM-254890 treatment exhibited no significant effect on Lgmn-induced ER Ca^2+^ release in these cells (Fig. 5A). These findings indicate that the signaling mechanism of µ-OR1 in response to Lgmn operates through G_αi_, rather than G_βϒ_ or G_q._ In *+sh*µ-OR1* cells, YM-254890 completely abolished Lgmn-evoked ER Ca²⁺ release, while PTx and Gallein had no significant impact on this process (Fig. 5B), indicating that Lgmn-evoked PAR2-mediated ER Ca^2+^ release functions through G_αq_. For positive and negative controls, we used D,L-methadone and 2FLI that specifically stimulate µ-OR1 and PAR2, respectively, transduce their signals through G_αi_ in *+sh*PAR2* cells (but not through G_βϒ_ or G_q_; Fig. 5C) and G_q_ in *+sh*µ-OR1* cells (but not through G_αi_ or G_βϒ_; Fig. 5D), respectively [10].

Next, we sought to determine whether our findings in SEM B-ALL cells could be replicated in MOLT3 T-ALL cells. To achieve this, the cells were first pretreated with GB83 or CTAP to induce the PAR2 or µ-OR1 knockdown effect, followed by exposure to either PTx, Gallein, or YM-254890 prior to treatment with Lgmn. The cells were then analyzed via single-cell Ca²⁺ imaging to assess the extent of ER Ca²⁺ release. Keeping in line with our findings in SEM cells, pretreatment with GB83 alone in MOLT3 cells inhibited approximately 30.4% of Lgmn-induced ER Ca^2+^ release, whereas treatment of both GB83 and PTx eliminated the remaining ER Ca^2+^ release (Supplementary Fig. 2A), thereby recapitulating our observation in SEM B-ALL cells (Fig. 5A) in MOLT3 T-ALL cells. Conversely, treatment of both GB83 and Gallein or YM-254890 displayed no further impact on the remaining ER Ca^2+^ release (Supplementary Fig. 2B). In MOLT3 cells pretreated with CTAP, Lgmn-induced ER Ca^2+^ release was reduced approximately 81.9%, while pretreatment of both CTAP and Gallein had no further effect on the remaining ER Ca^2+^ release (Supplementary Fig. 2C). In contrast, pretreatment of both CTAP and YM-254890 removed the remaining Ca^2+^ release (Supplementary Fig. 2D), thereby recapitulating our observation in SEM B-ALL cells in MOLT3 T-ALL cells. Altogether, these results on MOLT3 cells further reinforce the µ-OR1-G_αi_ and PAR2-G_αq_ signaling pathways in Lgmn-induced ER Ca^2+^ release.

### Lgmn-induced µ-OR1-mediated ER Ca²^+^ release in ALL cells is negatively regulated by adenylate cyclase (AC), cAMP and cAMP-dependent protein kinase (PKA)

**Fig. 6:**
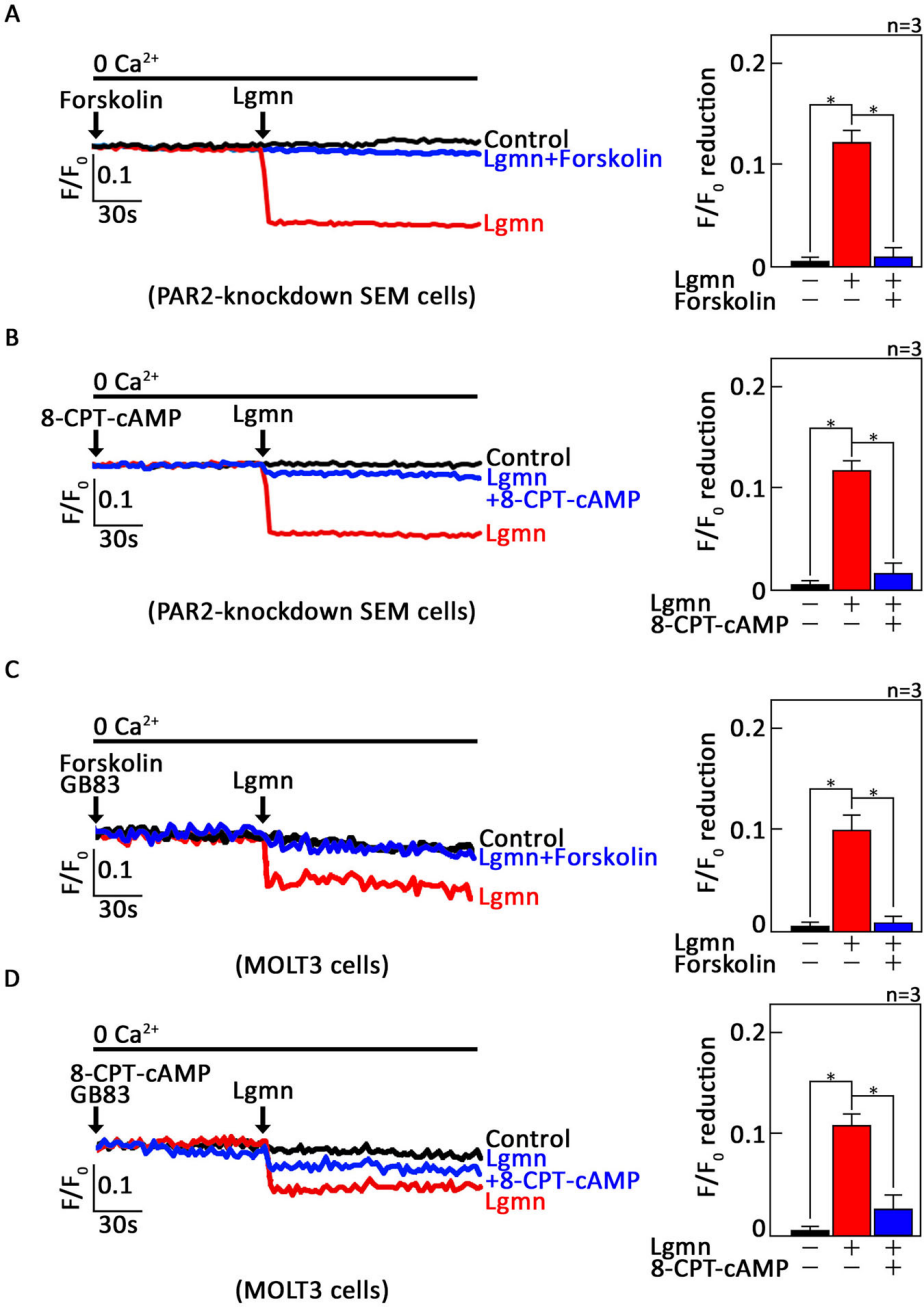
Lgmn-induced µ-OR1-mediated ER Ca^2+^ release is attenuated following stimulation of AC with forskolin or treatment with exogenous 8-CPT-cAMP. **A**, **B** *+sh*PAR2* cells, preloaded with Mag-Fluo-4 AM, were treated (or not) with forskolin (**A**) or 8-CPT-cAMP (**B**), before being exposed to Lgmn. **C** & **D**. MOLT3 cells, also preloaded with Mag-Fluo-4 AM, were treated (or not) with GB83 to inhibit PAR2. Following this, the cells were exposed to forskolin (**C**) or 8-CPT-cAMP (**D**), followed by Lgmn. Single-cell Ca^2+^ imaging was then conducted to evaluate ER Ca^2+^ release. The panels on the left (**A**–D) illustrate the average Ca2+ tracing recorded every 2 s from 20 individual cells, both before and after Lgmn treatment. The data presented are from one of three independent experiments (*n* = 3), yielding similar results. The chart on the right depicts the differences in ER Ca^2+^ release after Lgmn treatment. An *F*/*F*_0_ value of 30 s post-addition of Lgmn was utilized to determine *F*/*F*_0_ reduction in the right panels of (**A**–**D**). Values are presented as means ± SEM from three independent experiments, with *p < 0.05 statistical significance; “N.S.” denotes not significant.

We next aimed to determine whether G_αi_-driven ER Ca²⁺ release, mediated by µ-OR1 following Lgmn treatment, involves AC and cAMP. For this purpose, *+sh*PAR2* preloaded with Mag-Fluo-4 AM were treated with either the AC activator forskolin [31] or the exogenous cell-permeable cAMP analog, 8-CPT-cAMP, prior to exposure to Lgmn. This setup allowed for analysis via single-cell Ca²⁺ imaging to assess ER Ca²⁺ release. As depicted in Fig. 6A, both forskolin and 8-CPT-cAMP completely inhibited Lgmn-induced ER Ca²⁺ release in *+sh*PAR2* cells, indicating that AC and cAMP play a role in this process. Subsequently, we pretreated MOLT3 cells with GB83 to inhibit the PAR2 pathway, followed by treatment with forskolin or 8-CPT-cAMP, and then exposed the cells to Lgmn, analyzing them via single-cell Ca²⁺ imaging. Notably, our observations in MOLT3 T-ALL cells paralleled those noted in SEM cells. Thus, these findings further strengthen the role of AC and cAMP in this process.

**Fig. 7:**
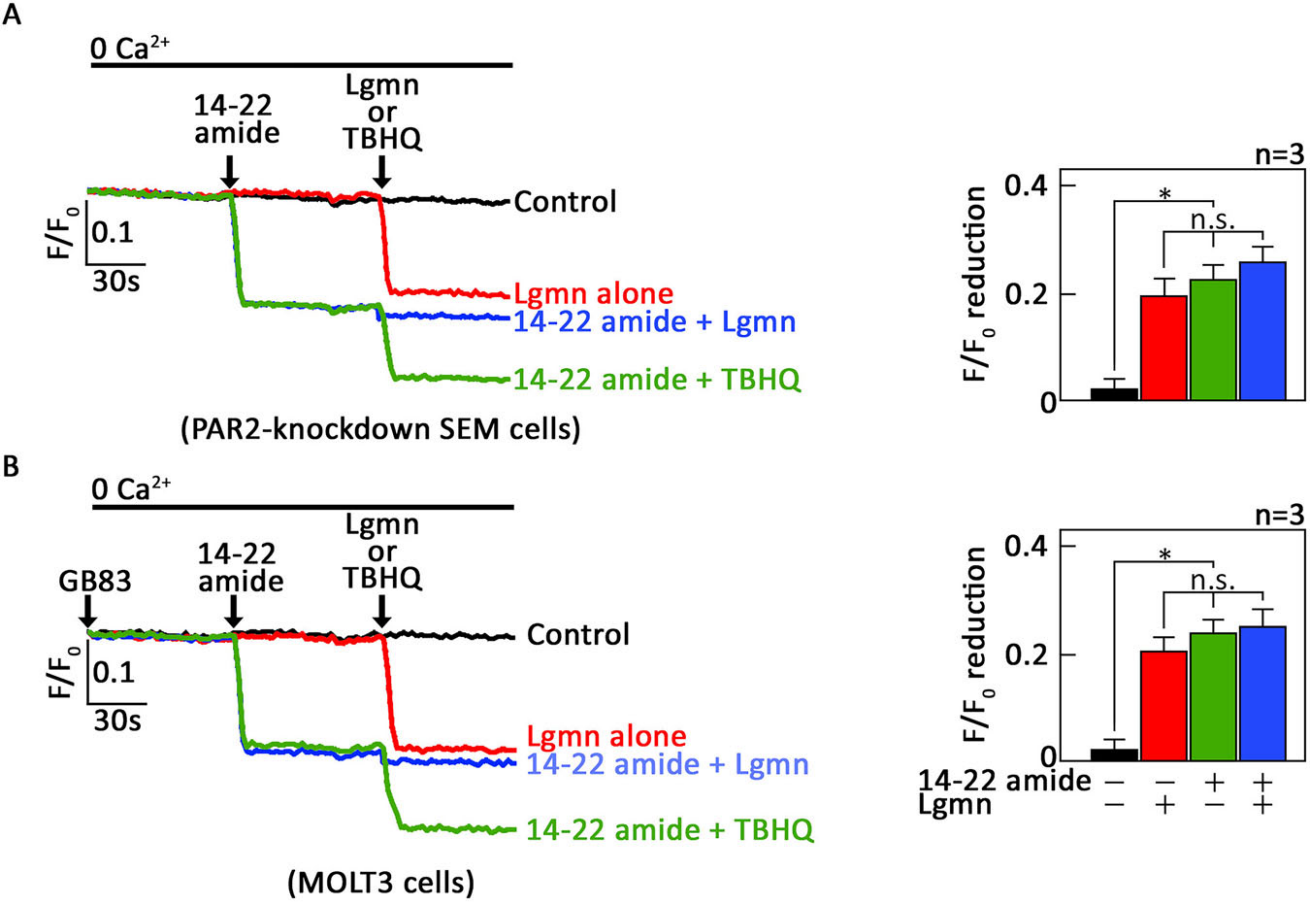
14–22 amide alone is sufficient to stimulate Lgmn-induced µ-OR1-mediated ER Ca^2+^ release. **A** *+sh*PAR2* cells, preloaded with Mag-Fluo-4 AM, were treated (or not) with 14–22 amide, before being exposed to Lgmn or TBHQ. **B** MOLT3 cells, also preloaded with Mag-Fluo-4 AM, were treated with GB83 to inhibit PAR2. Following this, the cells were exposed (or not) to 14–22 amide, before being treated with Lgmn or TBHQ. Single-cell Ca^2+^ imaging was then conducted to evaluate ER Ca^2+^ release. The panels on the left in (**A**, **B**) illustrate the average Ca^2+^ tracing recorded every 2 s from 20 individual cells, both before and after the treatment with 14–22 amide, Lgmn, or TBHQ. The data presented are from one of three independent experiments (*n* = 3), yielding similar results. The chart on the right depicts the differences in ER Ca^2+^ release after 14–22 amide, Lgmn or TBHQ treatment. An *F*/*F*_0_ value of 30 s post-addition of Lgmn was utilized to determine *F*/*F*_0_ reduction in the right panels of (**A**, **B**). Values are presented as means ± SEM from three independent experiments, with *p < 0.05 statistical significance; “N.S.” denotes not significant.

cAMP regulates intracellular Ca²⁺ concentrations through PKA-mediated inhibitory phosphorylation on PLCβ3 [14, 32, 33]. This suggests that activation of µ-OR1 by Lgmn, which triggers release of Ca²⁺ from the ER, may also involve the inhibition of PKA. If this is correct, we expect that treatment with the myristylated, cell-permeable, PKA-specific inhibitor 14–22 amide (14–22 amide) [34] alone in *+sh*PAR2* cells will lead to ER Ca²⁺ release. Furthermore, pretreatment with 14–22 amide should not result in an additional increase in Lgmn-induced ER Ca²⁺ release. As demonstrated in Fig. 7A, the administration of 14–22 amide alone successfully evoked ER Ca²⁺ release (illustrated in green and blue tracings). Following this treatment, exposure to Lgmn did not elicit any further ER Ca²⁺ release (also marked in blue), suggesting that the µ-OR1-G_αi_-AC-cAMP-dependent mechanism of Lgmn-induced ER Ca²⁺ release involves PKA inhibition. This pathway appears capable of bypassing the upstream Lgmn-µ-OR1-AC-cAMP signaling steps. Additionally, treatment with the ER Ca²⁺ pump inhibitor TBHQ resulted in further Ca²⁺ release, confirming the viability of the cells throughout the analysis (Fig. 7). Subsequently, we pretreated MOLT3 cells with GB83 to achieve the PAR2 knockdown effect, followed by treatment with 14–22 amide, before exposing them to Lgmn. Notably, our observations in MOLT3 T-ALL cells mirrored those noted in SEM cells. Therefore, our findings suggest the AC-cAMP-PKA cascade operates downstream of the µ-OR1-G_αi_ signaling pathway.

### Lgmn-induced µ-OR1-mediated ER Ca²^+^ release is associated with a reduction in the phosphorylation of PLCβ3 at Ser1105 and BAD at Ser118, both of which are regulated by PKA

**Fig. 8:**
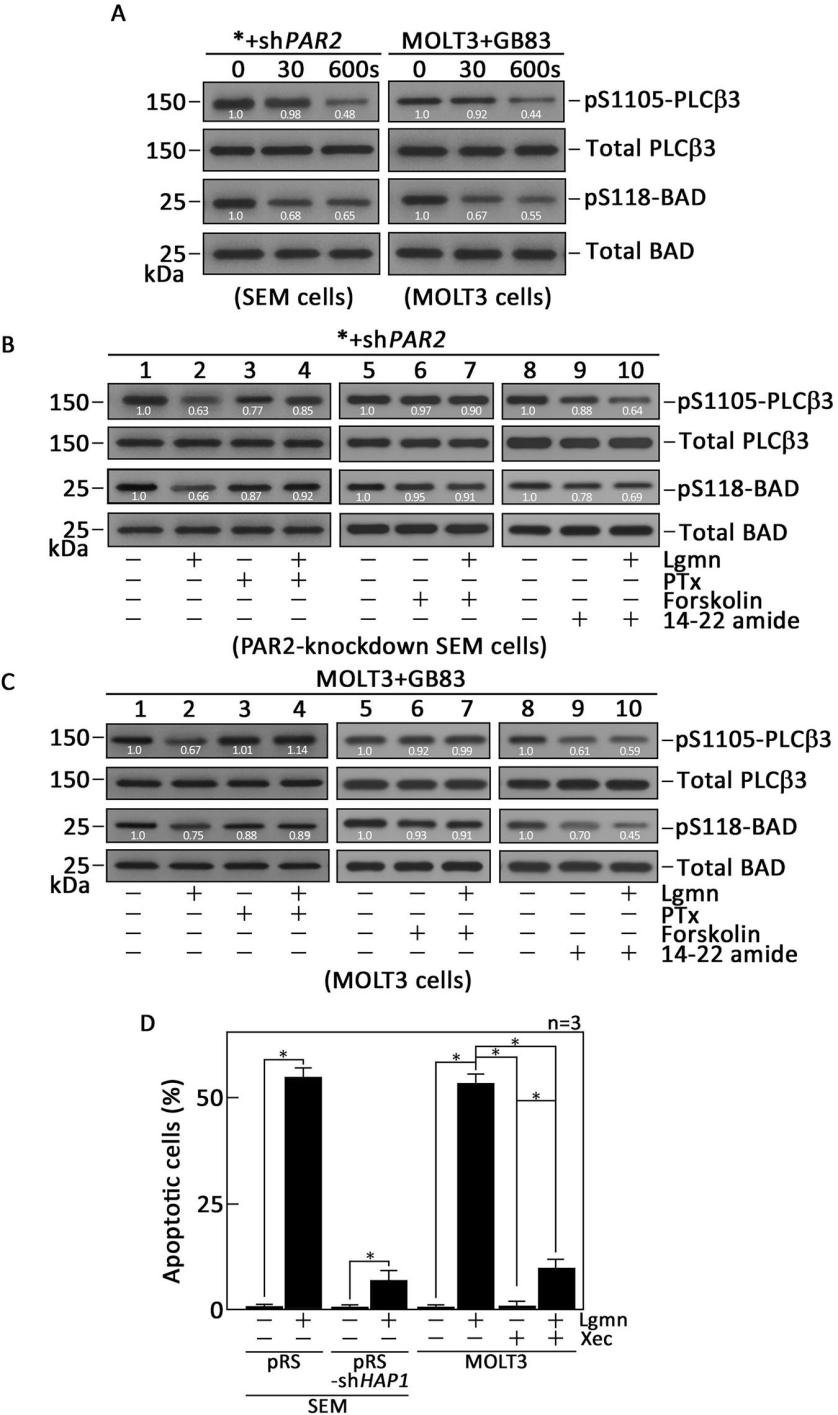
Lgmn-induced µ-OR1-mediated ER Ca^2+^ release is linked to decreased phosphorylation of PLCβ3 at Ser1105 and BAD at Ser118. **A** Lysates from *+sh*PAR2* (left panel), and MOLT3 (right panel) cells pretreated with GB83, were exposed to Lgmn for the specified durations. Lysates from *+sh*PAR2* (**B**), and MOLT3 (**C**) cells pretreated with GB83, were exposed to Lgmn for 600 s. The cell lysates underwent SDS‒PAGE and subsequent immunoblotting for pSer1105-PLCβ3 and total PLCβ3, as well as pSer118-BAD and total BAD. Numbers beneath the pSer1105-PLCβ3 and pSer118-BAD bands indicate the relative intensity ratios of the pSer1105-PLCβ3 or pSer118-BAD to total PLCβ3 or BAD bands, respectively, with values at time 0 normalized to 1. **D** Lgmn instigates ALL cell apoptosis. SEM B-ALL and MOLT3 T-ALL cells, treated with Lgmn for 16 h, were double-stained with FITC-labeled Annexin V and PI, followed by flow cytometry analysis. The SEM cells, which were stably transduced with retrovirus carrying pRS-*HAP1* shRNA [9], and MOLT3 cells pretreated with Xestospongin C (XeC) [39], an inhibitor of ER Ca^2+^, served as controls. The percentages of Annexin V + /PI+ cells in Q2, representing late apoptotic cell populations, and Annexin V + /PI− cells in Q4, representing early apoptotic cells, were quantified. Values are expressed as means ± SEM of three independent experiments (*n* = 3). *p < 0.05. N.S., not significant.

**Fig. 9:**
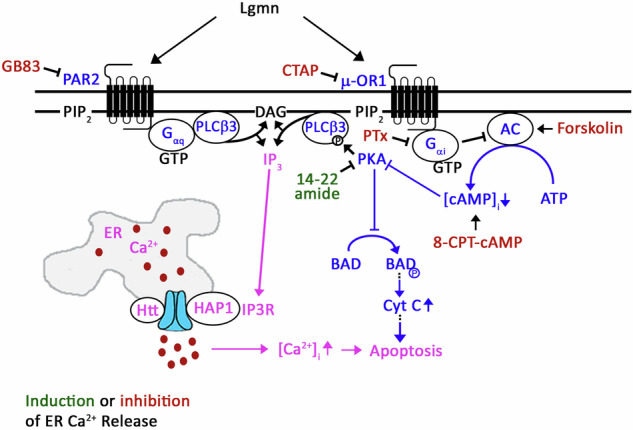
Proposed model illustrating Lgmn-induced ER Ca^2+^ release and apoptosis in ALL cells. Lgmn instigates ER Ca^2+^ release through two separate µ-OR1-G_αi_ and PAR2-G_αq_ pathways (in blue). The G_αq_ directly stimulates PLCβ3 [35–37], which leads to ER Ca²⁺ release via the IP3/IP3R pathway (shown in pink). Conversely, the µ-OR1 pathway entails G_αi_-mediated inhibition of adenylyl cyclase (AC), resulting in a decrease in [cAMP]_i_ (Supplementary Figure 5). This decline in [cAMP]_i_ subsequently reduces PKA activity, causing lower levels of pSer1105-PLC3. As a result, this encourages ER Ca²⁺ release through the IP3/IP3R pathway (also in pink). Moreover, the µ-OR1 pathway is associated with decreased PKA-mediated phosphorylation of BAD at Ser118 (in blue). This process, together with the intracellular Ca²⁺ imbalance triggered by ER Ca²⁺ release, ultimately contributes to apoptosis in ALL cells. Small molecular agonists and antagonists, depicted in red, inhibit ER Ca^2+^ release, while 14–22 amide, shown in green, induces ER Ca^2+^ release.

The G_αq_ subunit directly activates PLCβ3 [35–37], thereby stimulating PLCβ3-mediated ER Ca²⁺ release through the IP3/IP3R pathway. However, PKA is known to phosphorylate PLCβ3 at Ser1105, and the downregulation of this phosphorylation enhances PLCβ3-mediated ER Ca²⁺ release [38]. Consequently, we began by investigating whether the Lgmn-µ-OR1-G_αi_-AC-cAMP-PKA signaling axis is responsible for downregulating the phosphorylation of PLCβ3 at Ser1105, which in turn triggers the PLCβ3-mediated ER Ca²⁺ release. To do this, we analyzed lysates from *+sh*PAR2* cells and MOLT3 cells that had been pretreated with GB83 and then treated with Lgmn for designated periods. These lysates were resolved using SDS-PAGE and immunoblotted for pSer1105-PLCβ3. As demonstrated in Figs. 8A, 8B (see uncropped blots in Supplemental Material), Lgmn significantly decreased the level of pSer1105-PLCβ3, which corresponded with the observed ER Ca²⁺ release upon Lgmn treatment. Pretreatment of *+sh*PAR2* cells with PTX or forskolin caused a modest change in the Lgmn-induced PLCβ3 phosphorylation at Ser1105 (Fig. 8B), consistent with the lack of Lgmn-induced ER Ca²⁺ release under PTX or forskolin treatment (Figs. 5A and 6A). Since cAMP stimulates PKA, leading to the phosphorylation of BAD at Ser118 and disrupting the interaction between BCL2 and BAD, which in turn enhances BCL2’s antiapoptotic activity [15, 16], we also examined whether Lgmn negatively impacts BAD phosphorylation at Ser118. Figures 8A, 8B display that, similar to pSer1105-PLCβ3, the level of pSer118-BAD was reduced following Lgmn treatment. Moreover, pretreatment with PTX or forskolin caused no or a minor impact on Lgmn-induced BAD phosphorylation at Ser118 (Fig. 8B). We also assessed the impact of PKA inhibition by 14–22 amide on PLCβ3 stimulation and BAD phosphorylation at Ser118 in *+sh*PAR2* cells. As shown in Fig. 8B, treatment with Lgmn or 14–22 amide significantly decreased the phosphorylation of PLCβ3 at Ser1105 and BAD at Ser118, confirming that PKA mediates this phosphorylation. Notably, our observation that 14–22 amide alone led to a significant decrease in PLCβ3 phosphorylation at Ser1105 aligns with our finding that it also induced ER Ca^2+^ release. This suggests that Lgmn-induced µ-OR1-mediated ER Ca²⁺ release occurs via the G_αi_-AC-cAMP-PKA-PLCβ3 axis (Fig. 9). Importantly, these observations in *+sh*PAR2* cells were similar to those noted in MOLT3 T-ALL cells pretreated with GB83. Thus, our results imply that the AC-cAMP-PKA-PLCβ3 and the AC-cAMP-PKA-BAD pathways operate downstream of the µ-OR1-G_αi_ following Lgmn treatment.

### Lgmn induces apoptotic cell death in ALL cells

The disruption of intracellular Ca^2+^ homeostasis, resulting from ER Ca^2+^ release, has been associated with the death of ALL cells [9]. To investigate whether Lgmn induces apoptosis, SEM B-ALL and MOLT3 T-ALL cells were treated with Lgmn for 16 h, followed by double-stained with FITC-labeled Annexin V and PI. Flow cytometry analysis was conducted to evaluate apoptotic cell death. As demonstrated in Fig. 8D and Supplementary Fig. 4, Lgmn indeed triggered apoptotic cell death in ALL cells. This effect was reversed in SEM cells that lack HAP1, a vital component of the ternary HAP1-Htt-IP3R Ca^2+^ channel required for ER Ca^2+^ release [9]. As a result, these cells did not experience Lgmn-induced ER Ca^2+^ release (Supplementary Fig. 3). Furthermore, MOLT3 cells pretreated with Xestospongin C (XeC) [39], an inhibitor of ER Ca^2+^ (Supplementary Fig. 3C), also reversed this effect. This establishes a clear link between the disruption of intracellular Ca²⁺ balance due to ER Ca²⁺ release and the subsequent apoptosis.

## Discussion

Parasitic protists such as *Blastocystis* and *Trichomonas* are responsible for infections and illnesses affecting both humans and animals globally. Recent statistics indicate that these harmful protozoa impact over 1.5 billion individuals and result in approximately 300,000 fatalities each year [40]. *Blastocystis* inhabits the intestinal tract and is associated with various gastrointestinal disorders, including diarrhea, bleeding, chronic bowel inflammation, malabsorption issues, and irritable bowel syndrome (IBS) [6]. In contrast, *Trichomonas* is mostly found in the urogenital tract and oral cavity, leading to a sexually transmitted infection (STI) known as Trichomoniasis. This infection is characterized by itching in the genital area, a foul-smelling thin vaginal discharge, painful urination, and discomfort during sexual intercourse, while also increasing the risk of contracting HIV/AIDS. Lgmn is a major virulence factor for these protozoan parasites [40, 41], playing a role in the invasion and destruction of host cells. Nevertheless, the exact targets of Lgmn and the specific molecular pathways through which it produces its cytotoxic effects on host cells are still unclear.

Recently, Lgmn has been shown to facilitate the mobilization of intracellular Ca²⁺ in HEK293 human embryonic kidney cells by specifically targeting a GPCR PAR2, particularly its N-terminal residue, N_30_-R_31_ [7]. This same site is also acted upon by L-asparaginase [8], a key chemotherapeutic drug employed to eliminate ALL cells. In doing so, L-asparaginase induces IP3R-mediated Ca^2+^ release from the ER by targeting PAR2 and another GPCR µ-OR1 [10]. This disrupts the balance of intracellular Ca^2+^ in ALL cells and activates the Ca^2+^-regulated apoptotic pathway [10] involving calpain-1, Bid, cyt C, and caspase-3 and -12 [9]. The use of BAPTA-AM for Ca^2+^ chelation, which counteracts the apoptotic effects of L-asparaginase in ALL cells, establishes a direct connection between the rise in [Ca^2+^]_i_ caused by L-asparaginase and the apoptosis of ALL cells [9]. This paradigm, in which L-asparaginase targets two distinct GPCRs, PAR2 and µ-OR1, to induce the death of ALL cells may contribute to resolving ongoing questions regarding the molecular mechanisms by which Lgmn applies its cytotoxic effects on host cells. We embarked on this investigation to determine whether Lgmn, an asparaginyl endopeptidase (AEP), is capable of inducing Ca²⁺ release from the ER in HEK293 cells. If this proved to be the case, we aimed to further examine whether Lgmn could also trigger ER Ca²⁺ release in these cells through the activation of µ-OR1. The selection of µ-OR1 was based on the understanding that GPCRs account for up to 5% of mammalian genomes and play a major role as upstream regulators in numerous signal transduction pathways. Our results indeed demonstrated that Lgmn prompts Ca²⁺ release from the ER via the activation of both PAR2 and µ-OR1, a finding replicated in ALL cells. Utilizing both PAR2- and µ-OR1-knockdown SEM cells, along with specific antagonists and agonists for each GPCR, enabled us to validate our conclusions. This encourages us to further explore more effective therapeutic strategies for ALL, particularly by investigating Lgmn as a promising therapeutic option for this condition.

Next, we explore the contributions of PAR2 and µ-OR1 for Lgmn-induced ER Ca²⁺ release in ALL cells. By utilizing two representative B-ALL and T-ALL cells, as well as the µ-OR1 antagonist CTAP and the PAR2 antagonist GB83, either individually or together, we established that µ-OR1 plays a significant role in Lgmn-mediated ER Ca²⁺ release. In contrast, the impact of PAR2 seems to be comparatively smaller across both B- and T-ALL cell subtypes. The notion that Lgmn triggers ER Ca^2+^ release through µ-OR1 and PAR2 was reinforced by our findings, which indicate that pretreating B- and T-ALL cells with CTAP and GB83 entirely inhibits ER Ca^2+^ release.

Upon activation, GPCRs, including µ-OR1 and PAR2, change their conformation, which facilitates the exchange of GDP for GTP in specific G-protein subsets to transmit signals. To demonstrate that Lgmn-induced ER Ca^2+^ release through µ-OR1 and PAR2 involves G_αi_ and G_αq_, respectively, we utilized G-protein selective inhibitors along with PAR2- and µ-OR1-knockdown SEM cells. These results align with earlier studies indicating that G_αi_ functions as a downstream effector for µ-OR1 activation in ALL cells [10, 13], and G_αq_ operates for PAR2 in ALL [10] and HEK293 [42] cells. It is also expected that the level at which PTx, a G_αi_ inhibitor, reduced Lgmn-induced ER Ca^2+^ release in MOLT3 cells (Supplementary Fig. 2) corresponded to the extent of inhibition by CTAP in MOLT3 cells (Fig. 4D), suggesting that Lgmn triggers ER Ca^2+^ release mainly via the µ-OR1-G_αi_ pathway.

Given that G_αi_-mediated inhibition of adenylyl cyclase (AC) leads to a reduction in [cAMP]_i_, which subsequently downregulates PKA activity, we explored the roles of AC and cAMP within the µ-OR1-G_αi_ signaling axis in PAR2-knockdown SEM cells. Our results indicate that activation of AC with forskolin, together with exogenous 8-CPT-cAMP, negated Lgmn-induced ER Ca^2+^ release. This supports our conclusion that µ-OR1-mediated ER Ca^2+^ release relies on G_αi_, as Lgmn lowers [cAMP]_i_ through G_αi_-mediated inhibition of AC activity. Furthermore, our observation that the 14–22 amide alone elicited ER Ca^2+^ release, and that additional Lgmn treatment did not provoke any further release, suggests that the inhibition of PKA activity is integral to Lgmn-induced µ-OR1-mediated ER Ca^2+^ release.

We subsequently investigated whether PLCβ3 and BAD act as downstream effectors of the µ-OR1-G_αi_-AC-cAMP-PKA axis. Our findings indicated that Lgmn triggered a PKA-mediated downregulation of PLCβ3 phosphorylation at Ser1105. Furthermore, pretreatment with PTx or forskolin did not affect the levels of pSer1105-PLCβ3 following Lgmn treatment. We also observed that Lgmn led to a reduction in pSer118-BAD levels. These results align with prior reports indicating that decreased PKA activity activates PLCβ3, resulting in IP3/IP3R-mediated ER Ca^2+^ release [10, 14]. Additionally, the lowered pSer118-BAD levels, due to decreased PKA activity, facilitate the formation of the BCL2-BAD complex, which counteracts the anti-apoptotic effects of BCL2. This interplay allows BAK and BAX to create pores in the outer mitochondrial membrane, leading to the release of cytochrome C, caspase cleavage, and subsequent apoptosis [17, 18].

As illustrated in Fig. 9, Lgmn triggers ER Ca^2+^ release through two distinct pathways: µ-OR1-G_αi_ and PAR2-G_αq_. The G_αq_ activates PLCβ3 [35–37], leading to ER Ca²⁺ release via the IP3/IP3R mechanism. Meanwhile, the µ-OR1 pathway decreases [cAMP]_i_ through G_αi_ inhibition of adenylyl cyclase (AC), resulting in a decrease in [cAMP]_i_. This decline in [cAMP]_i_ reduces PKA activity and levels of pSer1105-PLC3, which also promotes ER Ca²⁺ release. This pathway is linked to decreased PKA-mediated phosphorylation of BAD at Ser118. Consequently, the resulting intracellular Ca²⁺ imbalance contributes to apoptosis in ALL cells. Further research is needed to determine whether Lgmn targets µ-OR1 residues or whether other proteins are involved. Nonetheless, our findings suggest that inhibiting PKA activity with the 14–22 amide could disrupt the µ-OR1-AC-cAMP signaling axis, indicating that targeting PKA may be a viable therapeutic strategy for ALL.

## Materials and methods

### Materials

Fetal bovine serum (FBS) and penicillin/streptomycin were from Gibco (CA, USA). RPMI 1640, Mag-Fluo-4 AM (M14206), antibodies against µ-OR1 (PA5-26138) and phosphorylated Ser118 BAD (PA5-12550), an annexin V-FITC/propidium iodide (PI) apoptosis detection kit, and 4-(2-hydroxyethyl) piperazine-1-ethanesulfonic acid (HEPES: J63218.AK) were from ThermoFisher Scientific (Burlington, ON, Canada). D,L-methadone was from Market Drugs Medical Ltd (Edmonton, AB). CTAP (ab120680) and 8-CPT-cAMP (ab120424) were from Abcam (Toronto, ON). Pertussis toxin (PTx; P7208), GB83 (SML3529), 14–22 amide (myr; 476485), poly-L-ornithine (P3655), and 2,5-Di-tert-butylhydroquinone (TBHQ, 112976) were from Sigma-Aldrich (Oakville, ON, Canada). YM-254890 (29735) was from Cayman Chemical (Michigan, USA). Forskolin (S2449, 66575-29-9) was from Selleck Chemicals (TX, USA). Gallein (sc-202631), antibodies for total PLCβ3 (D-7), PAR2 (SAM11), actin (I-19) and BAD (C-7) were from Santa Cruz Biotechnology (Dallas, TX, USA). The antibody against phosphorylated Ser1105 PLC3 (2484) was obtained from Cell Signaling (Whitby, ON, Canada). 2-Furoyl-LIGRLO-amide (2fLI; 3015/1) was from Biotechne/Tocris (Hong Kong, China). Lgmn (2199-CY) was from Bio-techne (ON, Canada). Nitrocellulose membranes were from Pall Laboratory (ON, Canada).

### Generation of PAR2-knockdown HEK293T cells carrying hPAR2-N_30_A

PAR2-knockdown HEK293T cells and the pcDNA3.0 carrying hPAR2-N_30_A were generously provided by Dr. Brian Schmidt at New York University College of Dentistry. These PAR2-knockdown HEK293T cells were cultured in DMEM supplemented with 10% FBS and 1% penicillin/streptomycin. To transfect them with pcDNA3.0 carrying hPAR2-N_30_A, we utilized Lipofectamine 2000 (Thermo Fisher Scientific) and the cells were subsequently maintained in DMEM containing 10% FBS, 1% penicillin/streptomycin, and 50 μg/ml G418.

### Cell Culture

Established B-ALL (SEM [8–10, 22, 23] & POETIC2 [10, 20, 23, 25, 26]) and T-ALL (MOLT3 [23] & CEM [27]) cell lines were obtained from Dr. Aru Narendran at the University of Calgary and cultured in RPMI1640 at 37 °C in a 5% CO_2_ incubator. The culture media were supplemented with 10% FBS and 100 μg/ml penicillin/streptomycin. The generation of SEM cells that were stably transduced with lentivirus carrying sh*PAR2* or sh*µ-OR1* shRNA [10], as well as those stably transduced with retrovirus carrying pRS-*HAP1* shRNA [9], has been previously documented. Cells were tested for Mycoplasma contamination.

### Measurement of endoplasmic reticulum (ER) Ca^2+^ release

The ALL cells (0.2 × 10^6^) grown on 0.2 mg/ml poly-L-ornithine-coated 12 mm glass coverslips (VWR, PA, USA) were loaded with 5 μM Mag-Fluo-4 AM in a Ca^2+^-free Krebs-Ringer-Henseleit (KRH) buffer (composed of 25 mM HEPES pH 7.4, 125 mM NaCl, 5 mM KCl, 6 mM glucose, 1.2 mM MgCl₂, and 2 μM EGTA) containing 0.02% Pluronic F-127 and 0.1 mg/ml BSA for 30 min at 37 °C. Following this period, the coverslips were rinsed with the Ca^2+^-free KRH buffer to eliminate any residual Ca^2+^ dye, and then they were transferred to a 3.5 cm glass-bottomed plate containing 1 ml of the Ca^2+^-free KRH buffer. Ca^2+^ transients were monitored using the DMi8-Film confocal microscope (Leica Microsystems, IL, USA) at a magnification of 20x (*λ*_Ex_ = 495 _nm_ and *λ*_Em_ = 530 _nm_) in conjunction with the LASX imaging software (Leica Microsystems). After establishing stable baseline ER Ca^2+^ levels, the cells pretreated (or not) with 10 µM CTAP, 10 µM GB83, 0.1 µM PTx, 2 µM Gallein, 2 µM YM-254890, or 1.5 μM 14–22 amide (myr), 1 μM 8-CPT-cAMP, or 4 μM forskolin for the specified durations before being exposed to treatments with 20 ng/ml Lgmn, 5 µM 2-furoyl-LIGRLO-NH_2_ (2fLI), or 0.5 μg/ml D,L-methadone (and subsequently with 10 μM TBHQ). Average Ca^2+^ tracings were recorded every 2 s from 20 individual cells following treatment. Changes in ER fluorescence were evaluated as the signal-to-baseline ratio (SBR), calculated as the *F*/*F*_0_ ratio, where *F* represents the fluorescence value after stimulation and *F*_0_ indicates the basal or initial fluorescence.

### Western blot analysis

Lysates from cells (2 × 10^6^) that were pretreated or not pretreated with 0.1 µg/ml PTx, 4 μM forskolin or 2 µM 14–22 amide, followed by treatment (or no treatment) with 20 ng/ml Lgmn for the specified duration were resolved by SDS-PAGE and subsequently subjected to immunoblotting for PAR2, µ-OR1, pSer1105-PLCβ3, total PLCβ3, pSer118-BAD, and total BAD. Immunoreactive bands were visualized using enhanced chemiluminescence and detected using the Bio-Rad ChemiDoc Imager under the optimal exposure conditions. The ratios of pSer1105-PLCβ3 to total-PLCβ3 and pSer118-BAD to total BAD were calculated using the NIH ImageJ 1.61 software.

### Flow cytometry

Cells (0.5 × 10^6^), either pretreated or not pretreated with 0.1 µg/ml PTx, 4 μM forskolin, 2 µM 14–22 amide or 2 μM XeC were subsequently treated or left untreated with 20 ng/ml Lgmn for 16 h. Following this incubation, the cells were harvested, washed twice with 1× PBS, and double-stained with 2 μl each of Annexin V-FITC and propidium iodide. The cells were then analyzed using an Attune NxT flow cytometer (ThermoFisher Scientific, USA).

### Measurement of [cAMP]_i_

*+sh*PAR2* cells (1 × 10^6^ cells/well in 6-well plate) were pretreated (or not) with 10 μM CTAP for 30 min before being exposed to either 20 ng/ml Lgmn or 1.0 μg/ml D,L-methadone for 16 hours. Following treatment, the cell lysates were used to measure [cAMP]_i_ using the cyclic-AMP XP® assay kit (Cell Signaling, USA). Briefly, 50 μl of cell lysates were added to each well of a 96-well assay plate that was pre-coated with anti-cAMP XP® rabbit monoclonal antibody. The cAMP present in the samples competes with a fixed amount of HRP-linked cAMP for binding to the immobilized anti-cAMP XP® rabbit monoclonal antibody on the wells. After washing away unbound HRP-linked cAMP, 50 μl of the HRP substrate, TMB, was introduced. After 30 min at room temperature, the reaction was halted by adding 50 μl of sulfuric acid. The concentration of bound cAMP in the samples was then measured at 450 nm based on a cAMP standard curve.

### Statistical Analysis

The student’s unpaired, two-tailed t-test was performed at *p* < 0.05, which was considered statistically significant. For experiments involving more than two groups or treatments, one-way Analysis of Variance (ANOVA) with Tukey Honestly Significant Difference (HSD) post hoc tests was conducted to identify statistical differences between groups or treatments.

## Supplementary information

Supplementary Figure Legends

Supplementary Figure 1

Supplementary Figure 2

Supplementary Figure 3

Supplementary Figure 4

Supplementary Figure 5

Uncropped figures

## Data Availability

All data generated or analyzed during this study are included in this published article and its supplementary information files or uncropped data files.

## References

[1] Chen JM, Dando PM, Rawlings ND, Brown MA, Young NE, Stevens RA, et al. Cloning, isolation, and characterization of mammalian legumain, an asparaginyl endopeptidase. J Biol Chem. 1997;272:8090–8. 10.1074/jbc.272.12.8090. 9065484 10.1074/jbc.272.12.8090

[2] Haugen MH, Johansen HT, Pettersen SJ, Solberg R, Brix K, Flatmark K, et al. Nuclear legumain activity in colorectal cancer. PLoS One. 2013;8:e52980 10.1371/journal.pone.0052980. 23326369 10.1371/journal.pone.0052980 PMC3542341

[3] Wu B, Yin J, Texier C, Roussel M, Tan KS. Blastocystis legumain is localized on the cell surface, and specific inhibition of its activity implicates a pro-survival role for the enzyme. J Biol Chem. 2010;285:1790–8. 10.1074/jbc.M109.049064. 19915007 10.1074/jbc.M109.049064 PMC2804337

[4] Rawlings ND, Barrett AJ. Families of cysteine peptidases. Methods Enzymol. 1994;244:461–86. 10.1016/0076-6879(94)44034-4. 7845226 10.1016/0076-6879(94)44034-4 PMC7172846

[5] Barrett AJ, Rawlings ND. Families and clans of cysteine peptidases. Perspect Drug Discov Des. 1996;6:1–11. 10.1007/BF02174042. 32288275 10.1007/BF02174042 PMC7104565

[6] Wawrzyniak I, Texier C, Poirier P, Viscogliosi E, Tan KS, Delbac F, et al. Characterization of two cysteine proteases secreted by Blastocystis ST7, a human intestinal parasite. Parasitol Int. 2012;61:437–42. 10.1016/j.parint.2012.02.007. 22402106 10.1016/j.parint.2012.02.007

[7] Tu NH, Jensen DD, Anderson BM, Chen E, Jimenez-Vargas NN, Scheff NN, et al. Legumain induces oral cancer pain by biased agonism of protease-activated receptor-2. J Neurosci. 2021;41:193–210. 10.1523/JNEUROSCI.1211-20.2020. 33172978 10.1523/JNEUROSCI.1211-20.2020 PMC7786216

[8] Lee JK, Riabowol K, Wang X, Lee KY. L-asparaginase is a PAR2 N-terminal protease that unmasks the PAR2 tethered ligand. Cell Death Discov. 2025;11:152 10.1038/s41420-025-02467-z. 40195325 10.1038/s41420-025-02467-z PMC11977020

[9] Lee JK, Kang S, Wang X, Rosales JL, Gao X, Byun HG, et al. HAP1 loss confers l-asparaginase resistance in ALL by downregulating the calpain-1-Bid-caspase-3/12 pathway. Blood. 2019;133:2222–32. 10.1182/blood-2018-12-890236. 30819925 10.1182/blood-2018-12-890236 PMC6587669

[10] Lee JK, Kamran H, Lee KY. L-asparaginase induces IP3R-mediated ER Ca(2+) release by targeting micro-OR1 and PAR2 and kills acute lymphoblastic leukemia cells. Cell Death Discov. 2024;10:366 10.1038/s41420-024-02142-9. 39147734 10.1038/s41420-024-02142-9 PMC11327372

[11] Zhang J, Gu Y, Chen B. Mechanisms of drug resistance in acute myeloid leukemia. Onco Targets Ther. 2019;12:1937–45. 10.2147/OTT.S191621. 30881045 10.2147/OTT.S191621 PMC6417008

[12] Al-Hasani R, Bruchas MR. Molecular mechanisms of opioid receptor-dependent signaling and behavior. Anesthesiology. 2011;115:1363–81. 10.1097/ALN.0b013e318238bba6. 22020140 10.1097/ALN.0b013e318238bba6 PMC3698859

[13] Friesen C, Roscher M, Hormann I, Fichtner I, Alt A, Hilger RA, et al. Cell death sensitization of leukemia cells by opioid receptor activation. Oncotarget. 2013;4:677–90. 10.18632/oncotarget.952. 23633472 10.18632/oncotarget.952 PMC3742829

[14] Yue C, Dodge KL, Weber G, Sanborn BM. Phosphorylation of serine 1105 by protein kinase A inhibits phospholipase Cbeta3 stimulation by Galphaq. J Biol Chem. 1998;273:18023–7. 10.1074/jbc.273.29.18023. 9660757 10.1074/jbc.273.29.18023

[15] Lizcano JM, Morrice N, Cohen P. Regulation of BAD by cAMP-dependent protein kinase is mediated via phosphorylation of a novel site, Ser155. Biochem J. 2000;349:547–57. 10.1042/0264-6021:3490547. 10880354 10.1042/0264-6021:3490547 PMC1221178

[16] Virdee K, Parone PA, Tolkovsky AM. Phosphorylation of the pro-apoptotic protein BAD on serine 155, a novel site, contributes to cell survival. Curr Biol. 2000;10:R883 10.1016/s0960-9822(00)00843-5. 11114542 10.1016/s0960-9822(00)00843-5

[17] Gavathiotis E, Suzuki M, Davis ML, Pitter K, Bird GH, Katz SG, et al. BAX activation is initiated at a novel interaction site. Nature. 2008;455:1076–81. 10.1038/nature07396. 18948948 10.1038/nature07396 PMC2597110

[18] Westphal D, Dewson G, Czabotar PE, Kluck RM. Molecular biology of Bax and Bak activation and action. Biochim Biophys Acta. 2011;1813:521–31. 10.1016/j.bbamcr.2010.12.019. 21195116 10.1016/j.bbamcr.2010.12.019

[19] Rossi AM, Taylor CW. Reliable measurement of free Ca(2+) concentrations in the ER lumen using Mag-Fluo-4. Cell Calcium. 2020;87:102188. 10.1016/j.ceca.2020.102188. 32179239 10.1016/j.ceca.2020.102188 PMC7181174

[20] Lee J, Rosales JL, Byun HG, Lee KY. D,L-Methadone causes leukemic cell apoptosis via an OPRM1-triggered increase in IP3R-mediated ER Ca(2+) release and decrease in Ca(2+) efflux, elevating [Ca(2+)](i). Sci Rep. 2021;11:1009. 10.1038/s41598-020-80520-w. 33441856 10.1038/s41598-020-80520-w PMC7806773

[21] Furukawa A, Shinoda M, Kubo A, Honda K, Akasaka R, Yonehara Y, et al. Endothelin Signaling Contributes to Modulation of Nociception in Early-stage Tongue Cancer in Rats. Anesthesiology. 2018;128:1207–19. 10.1097/ALN.0000000000002139. 29461271 10.1097/ALN.0000000000002139

[22] Lee JK, Rosales JL, Lee KY. Requirement for ER-mitochondria Ca(2+) transfer, ROS production and mPTP formation in L-asparaginase-induced apoptosis of acute lymphoblastic leukemia cells. Front Cell Dev Biol. 2023;11:1124164. 10.3389/fcell.2023.1124164. 36895789 10.3389/fcell.2023.1124164 PMC9988955

[23] Kang SM, Rosales JL, Meier-Stephenson V, Kim S, Lee KY, Narendran A. Genome-wide loss-of-function genetic screening identifies opioid receptor mu1 as a key regulator of L-asparaginase resistance in pediatric acute lymphoblastic leukemia. Oncogene. 2017;36:5910–3. 10.1038/onc.2017.211. 28650467 10.1038/onc.2017.211 PMC5658664

[24] Muley MM, Reid AR, Botz B, Bölcskei K, Helyes Z, McDougall JJ. Neutrophil elastase induces inflammation and pain in mouse knee joints via activation of proteinase-activated receptor-2. Br J Pharmacol. 2016;173:766–77. 10.1111/bph.13237. 26140667 10.1111/bph.13237 PMC4742294

[25] Lee JK, Wang X, Wang J, Rosales JL, Lee KY. PKA inhibition kills L-asparaginase-resistant leukemic cells from relapsed acute lymphoblastic leukemia patients. Cell Death Discov. 2024;10:257 10.1038/s41420-024-02028-w. 38802344 10.1038/s41420-024-02028-w PMC11130271

[26] Kamran H, Lee JK, Lee KY. PKA inhibition is a central step in D,L-methadone-induced ER Ca(2+) release and subsequent apoptosis in acute lymphoblastic leukemia. Front Cell Dev Biol. 2024;12:1388745. 10.3389/fcell.2024.1388745. 38721527 10.3389/fcell.2024.1388745 PMC11076789

[27] Sandstrom PA, Buttke TM. Autocrine production of extracellular catalase prevents apoptosis of the human CEM T-cell line in serum-free medium. Proc Natl Acad Sci USA. 1993;90:4708–12. 10.1073/pnas.90.10.4708. 8506323 10.1073/pnas.90.10.4708 PMC46582

[28] Wu EH, Wong YH. Pertussis toxin-sensitive Gi/o proteins are involved in nerve growth factor-induced pro-survival Akt signaling cascade in PC12 cells. Cell Signal. 2005;17:881–90. 10.1016/j.cellsig.2004.11.008. 15763430 10.1016/j.cellsig.2004.11.008

[29] Sanz G, Leray I, Muscat A, Acquistapace A, Cui T, Rivière J, et al. Gallein, a Gbetagamma subunit signalling inhibitor, inhibits metastatic spread of tumour cells expressing OR51E2 and exposed to its odorant ligand. BMC Res Notes. 2017;10:541. 10.1186/s13104-017-2879-z. 29084601 10.1186/s13104-017-2879-z PMC5663063

[30] Uemura T, Kawasaki T, Taniguchi M, Moritani Y, Hayashi K, Saito T, et al. Biological properties of a specific Galpha q/11 inhibitor, YM-254890, on platelet functions and thrombus formation under high-shear stress. Br J Pharmacol. 2006;148:61–69. 10.1038/sj.bjp.0706711. 16520742 10.1038/sj.bjp.0706711 PMC1617042

[31] Insel PA, Ostrom RS. Forskolin as a tool for examining adenylyl cyclase expression, regulation, and G protein signaling. Cell Mol Neurobiol. 2003;23:305–14. 10.1023/a:1023684503883. 12825829 10.1023/A:1023684503883 PMC11530207

[32] Liu M, Simon MI. Regulation by cAMP-dependent protein kinease of a G-protein-mediated phospholipase C. Nature. 1996;382:83–87. 10.1038/382083a0. 8657310 10.1038/382083a0

[33] Ali H, Sozzani S, Fisher I, Barr AJ, Richardson RM, Haribabu B, et al. Differential regulation of formyl peptide and platelet-activating factor receptors: role of phospholipase Cβ3 phosphorylation by protein kinase A. J Biol Chem. 1998;273:11012–6. 10.1074/jbc.273.18.11012. 9556582 10.1074/jbc.273.18.11012

[34] Dalton GD, Smith FL, Smith PA, Dewey WL. Alterations in brain Protein Kinase A activity and reversal of morphine tolerance by two fragments of native Protein Kinase A inhibitor peptide (PKI). Neuropharmacology. 2005;48:648–57. 10.1016/j.neuropharm.2004.12.006. 15814100 10.1016/j.neuropharm.2004.12.006

[35] Smrcka AV, Hepler JR, Brown KO, Sternweis PC. Regulation of polyphosphoinositide-specific phospholipase C activity by purified Gq. Science. 1991;251:804–7. 10.1126/science.1846707. 1846707 10.1126/science.1846707

[36] Taylor SJ, Chae HZ, Rhee SG, Exton JH. Activation of the beta 1 isozyme of phospholipase C by alpha subunits of the Gq class of G proteins. Nature. 1991;350:516–8. 10.1038/350516a0. 1707501 10.1038/350516a0

[37] Waldo GL, Boyer JL, Morris AJ, Harden TK. Purification of an AlF4- and G-protein beta gamma-subunit-regulated phospholipase C-activating protein. J Biol Chem. 1991;266:14217–25. 1650351

[38] Zhong M, Murtazina DA, Phillips J, Ku CY, Sanborn BM. Multiple signals regulate phospholipase CBeta3 in human myometrial cells. Biol Reprod. 2008;78:1007–17. 10.1095/biolreprod.107.064485. 18322273 10.1095/biolreprod.107.064485 PMC2409063

[39] Solanes P, Heuzé ML, Maurin M, Bretou M, Lautenschlaeger F, Maiuri P, et al. Space exploration by dendritic cells requires maintenance of myosin II activity by IP3 receptor 1. EMBO J. 2015;34:798–810. 10.15252/embj.201489056. 25637353 10.15252/embj.201489056 PMC4369315

[40] Arguello-Garcia R, Carrero JC, Ortega-Pierres MG. Extracellular cysteine proteases of key intestinal protozoan pathogens-factors linked to virulence and pathogenicity. Int J Mol Sci. 2023;24. 10.3390/ijms241612850. 10.3390/ijms241612850 PMC10454693 37629029

[41] Leon-Felix J, Ortega-Lopez J, Orozco-Solis R, Arroyo R. Two novel asparaginyl endopeptidase-like cysteine proteinases from the protist Trichomonas vaginalis: their evolutionary relationship within the clan CD cysteine proteinases. Gene. 2004;335:25–35. 10.1016/j.gene.2004.03.002. 15194187 10.1016/j.gene.2004.03.002

[42] Hollenberg MD, Mihara K, Polley D, Suen JY, Han A, Fairlie DP, et al. Biased signalling and proteinase-activated receptors (PARs): targeting inflammatory disease. Br J Pharmacol. 2014;171:1180–94. 10.1111/bph.12544. 24354792 10.1111/bph.12544 PMC3952797

