# Supplementary material for: Lgmn targets two distinct GPCRs, PAR2 and µ-OR1, and induces cell death in acute lymphoblastic leukemia through an intracellular Ca²⁺ imbalance triggered by ER Ca²⁺ release
Source: Cell Death Discov. 2026 Mar 7;12:143. doi: 10.1038/s41420-026-03003-3 (PMC13039842; doi:10.1038/s41420-026-03003-3)
Supplement: Supplementary file 1 — Supplementary Figure Legends [file 41420_2026_3003_MOESM1_ESM.docx]

**Supplementary Figure Legends**

**Supplementary Figure 1. CTAP and GB83 selectively inhibit µ-OR1 and PAR2-mediated ER Ca²⁺ release, respectively, in SEM cells.** SEM cells, preloaded with Mag-Fluo-4 AM, were treated (or not) with CTAP (**A**) or GB83 (**B**), prior to exposure to D,L-methadone (**A**) or 2fLI (**B**). Single-cell Ca^2+^ imaging was then performed. The panels on the left in **A** & **B** display average Ca^2+^ traces recorded every 2 seconds from 20 individual cells, observed both before and after treatment with D,L-methadone (**A**) or 2fLI (**B**). The data presented are from one of three independent experiments (n=3), yielding similar results. The chart on the right illustrates the variations in ER Ca^2+^ release following the treatment of D,L-methadone (**A**) or 2fLI (**B**). An F/F_0_ value measured 30 seconds after the addition of D,L-methadone (**A**) or 2fLI (**B**) was utilized to assess F/F_0_ reduction for the right panels of **A** & **B**. The values are expressed as means ± SEM from three independent experiments, with *p<0.05 indicating statistical significance.

**Supplementary Figure 2. Lgmn also elicits ER Ca^2+^ release through the µ-OR1-G_αi_ and PAR2-G_αq_ pathways in MOLT3 T-ALL cells.** MOLT3 cells, preloaded with Mag-Fluo-4 AM, were treated (or not) with GB83 (**A** & **B**) or CTAP (**C** & **D**) to inhibit PAR2 or µ-OR1, respectively. Following this, the cells were exposed (or not) to either PTx (**A** & **C**), Gallein or YM-254890 (**B** & **D**) prior to Lgmn treatment. Single-cell Ca^2+^ imaging was conducted to evaluate ER Ca^2+^ release. The panels on the left (**A**-**D**) show the average Ca2+ tracing recorded every 2 seconds from 20 individual cells, both before and after Lgmn treatment. The data presented are from one of three independent experiments (n=3) that yield similar results. The chart on the right outlines the differences in ER Ca^2+^ release following Lgmn treatment. An F/F_0_ value, measured at 30 seconds post-addition of Lgmn, was used to determine F/F_0_ reduction in the right panels of **A-D**. Values are presented as means ± SEM from three independent experiments, with *p<0.05 statistical significance; “N.S.” denotes not significant.

**Supplementary Figure 3.** **The ability of** **Lgmn to induce ER Ca^2+^ release is inhibited (i) in SEM cells that lack HAP1, a component of the ternary HAP1-Htt-IP3R complex crucial for IP3-mediated ER Ca^2+^ release, and (ii) in MOLT3 cells pretreated XeC.** SEM cells (**A**) and those stably transduced with retrovirus carrying pRS-*HAP1* shRNA^9^ (**B**), as well as MOLT3 (**C**) were loaded with Mag-Fluo-4 AM. These cells were then treated (or not) with XeC. Following this, the cells were exposed to Lgmn, and single-cell Ca^2+^imaging was performed to measure ER Ca^2+^ release. Cells treated with L-asparaginase serve as positive controls. The left panels in **A**-**C** display average Ca^2+^ traces recorded every 2 seconds from 20 individual cells, both prior to and following Lgmn or L-asparaginase treatment. The data presented are from one of three independent experiments (n=3) that produced similar results. The chart on the right illustrates the differences in ER Ca^2+^ release following Lgmn or L-asparaginase treatment. An F/F_0_ value measured 30 seconds after the addition of Lgmn or L-asparaginase was used to assess the reduction in F/F_0_ for the right panels of **A** & **B**. The values are expressed as means ± SEM from three independent experiments, with *p<0.05 indicating statistical significance, while “N.S.” indicates not significant.

**Supplementary Figure 4.** Representative flow cytometry data for Figure 8D that show Annexin V+/PI+ cells in quadrant 2 (Q2) and Annexin V+/PI− cells in Q4. The percentages of cells in each quadrant are also shown.

**Supplementary Figure 5. Treatment with Lgmn in PAR2-knockdown SEM cells led to a reduction in [cAMP]_i_.** *+sh*PAR2* cells, either pretreated with CTAP or not, were treated with Lgmn for 16 hours after which [cAMP]_i_ was measured. The results are presented as mean ± SEM from three independent experiments (n=3). *p<0.05; n.s., not significant. Cells treated with D,L-methadone or CTAP serve as positive and negative controls.
