# Supplementary figures and images for: Lgmn targets two distinct GPCRs, PAR2 and µ-OR1, and induces cell death in acute lymphoblastic leukemia through an intracellular Ca²⁺ imbalance triggered by ER Ca²⁺ release
Source: Cell Death Discov. 2026 Mar 7;12:143. doi: 10.1038/s41420-026-03003-3 (PMC13039842; doi:10.1038/s41420-026-03003-3)

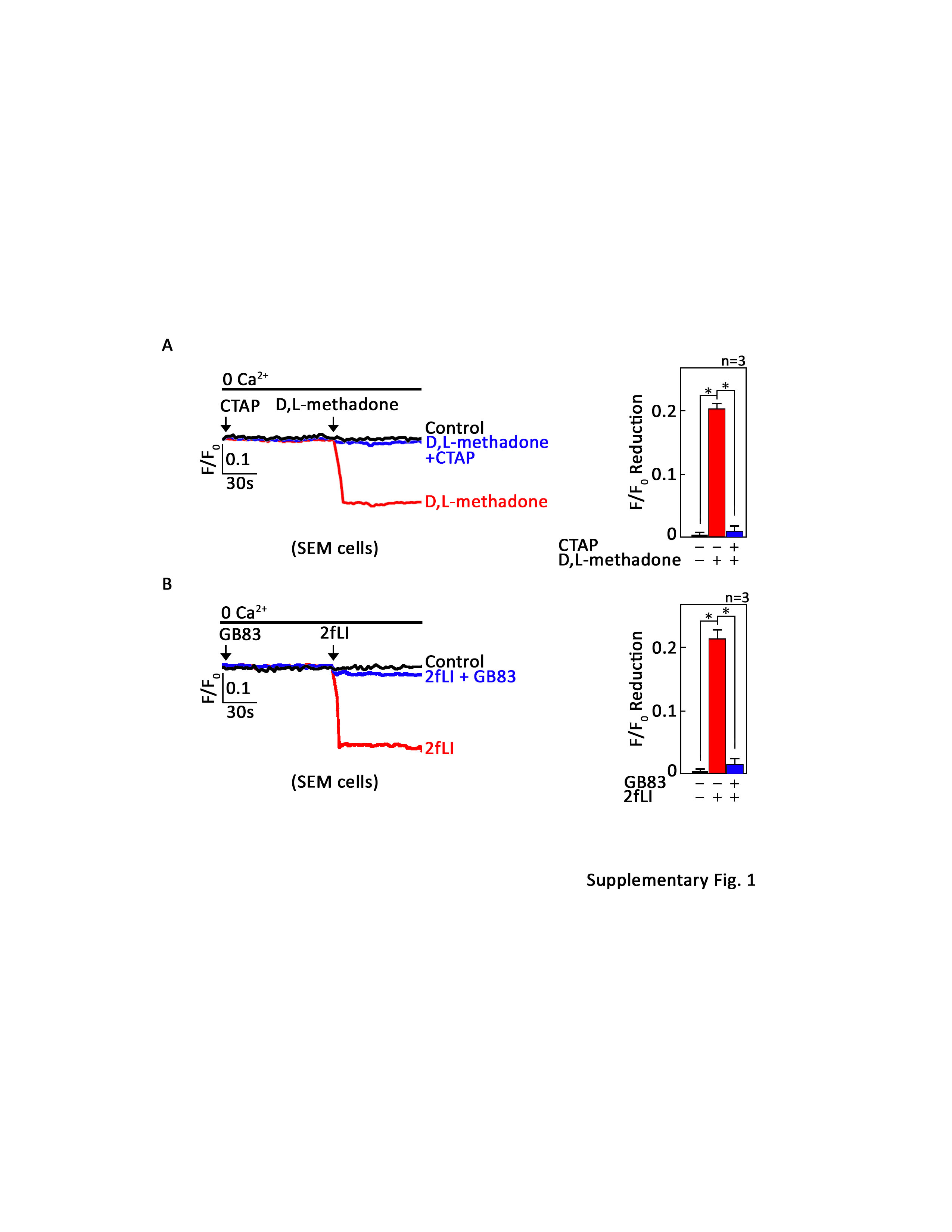

Supplement: Supplementary file 2 — Supplementary Figure 1 [file 41420_2026_3003_MOESM2_ESM.jpg]

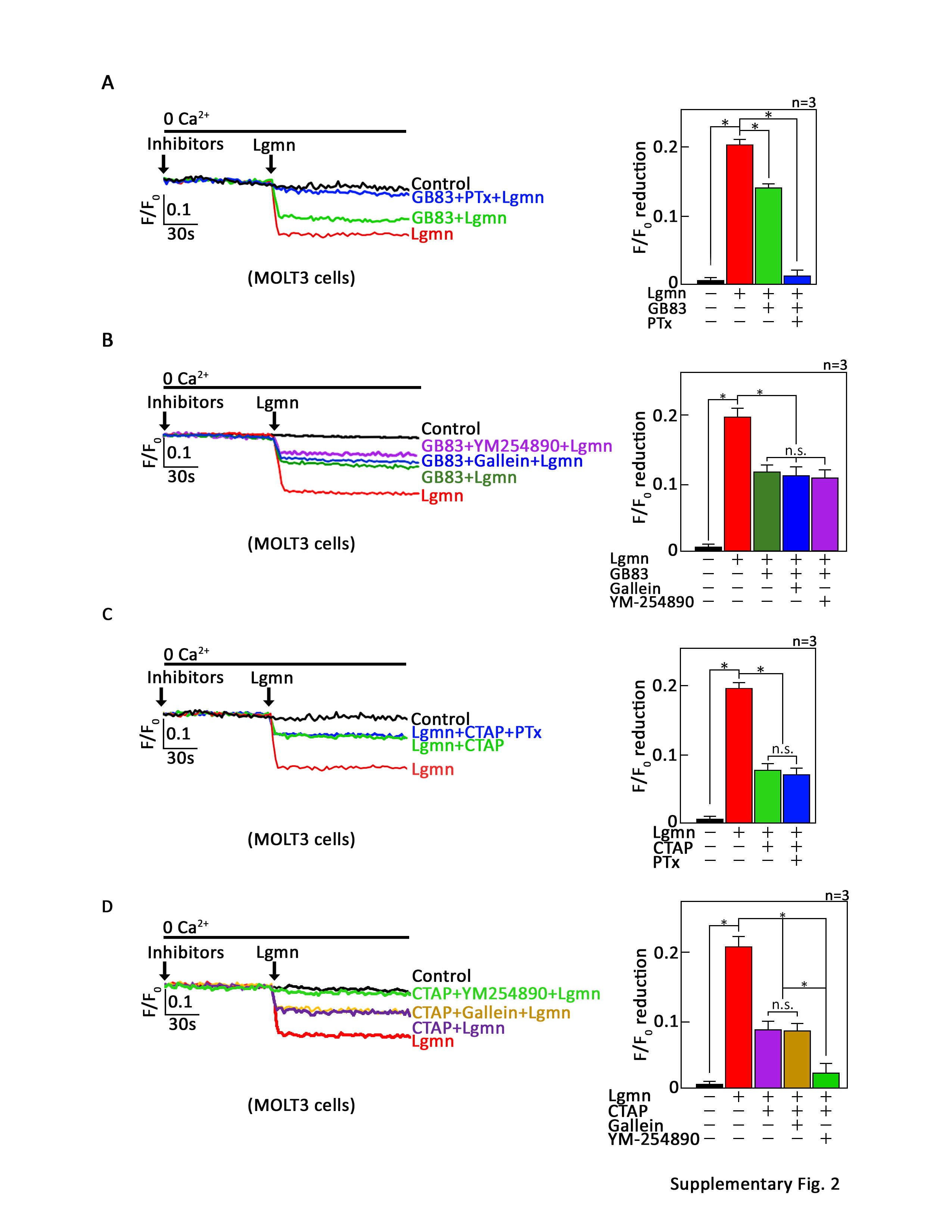

Supplement: Supplementary file 3 — Supplementary Figure 2 [file 41420_2026_3003_MOESM3_ESM.jpg]

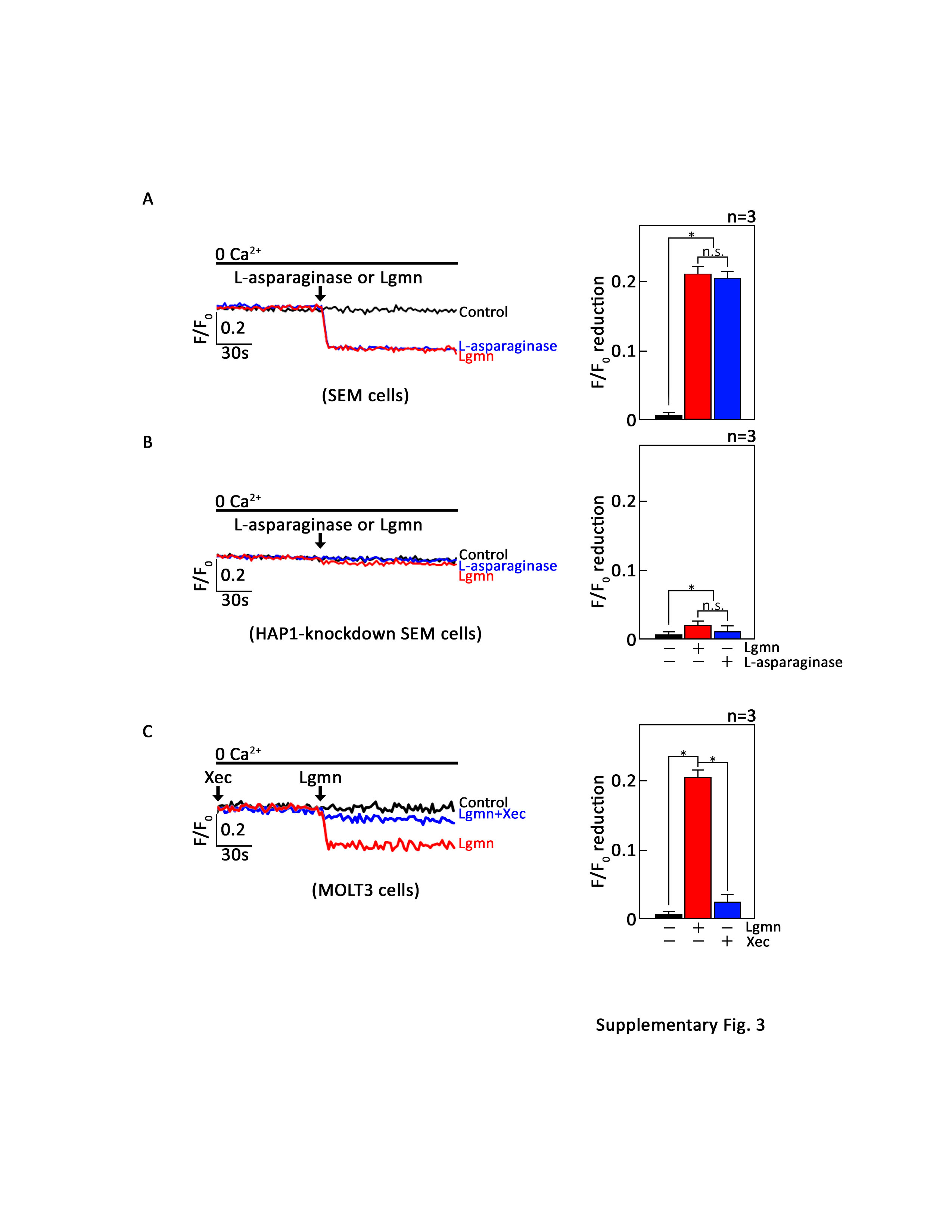

Supplement: Supplementary file 4 — Supplementary Figure 3 [file 41420_2026_3003_MOESM4_ESM.jpg]

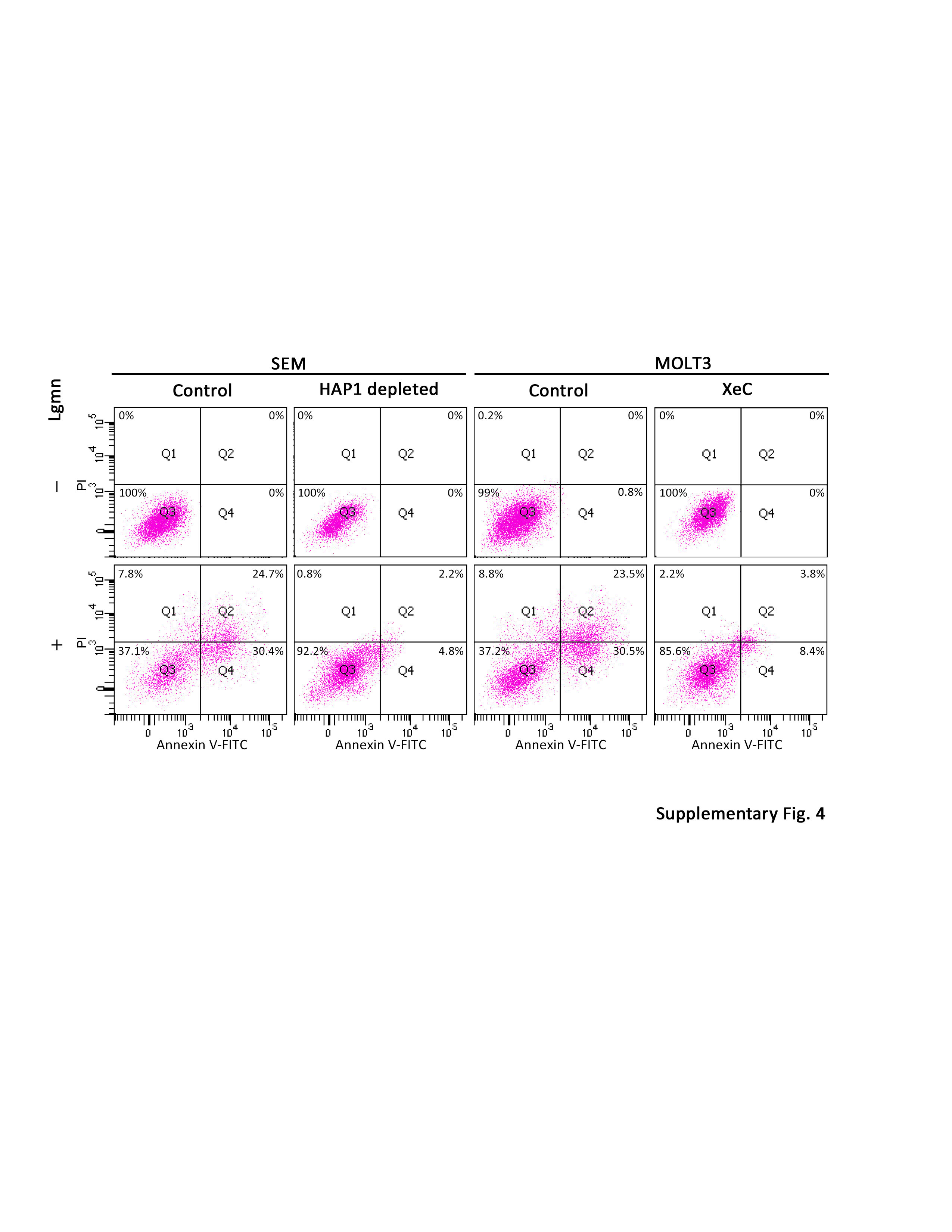

Supplement: Supplementary file 5 — Supplementary Figure 4 [file 41420_2026_3003_MOESM5_ESM.jpg]

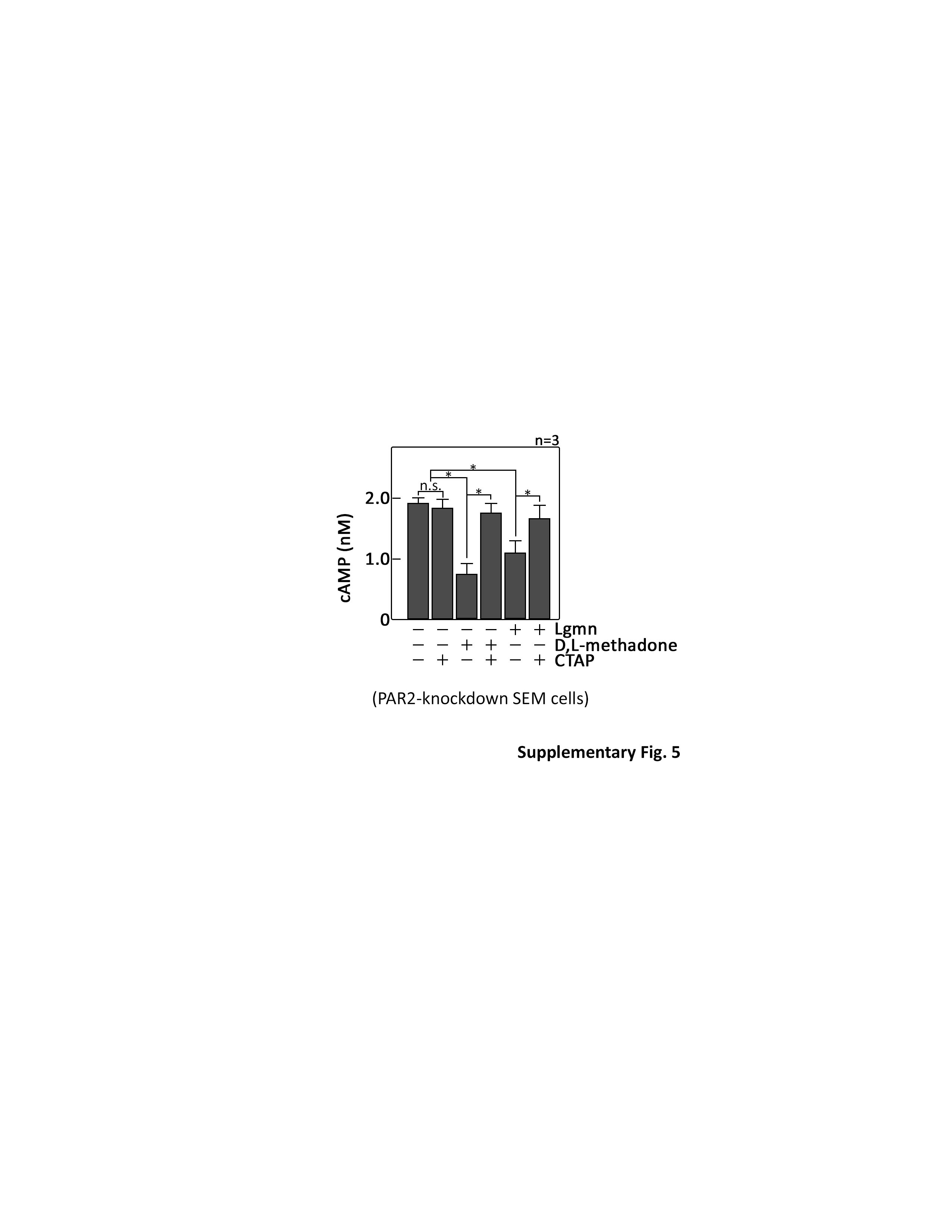

Supplement: Supplementary file 6 — Supplementary Figure 5 [file 41420_2026_3003_MOESM6_ESM.jpg]
