# Supplementary material for: Lgmn targets two distinct GPCRs, PAR2 and µ-OR1, and induces cell death in acute lymphoblastic leukemia through an intracellular Ca²⁺ imbalance triggered by ER Ca²⁺ release
Source: Cell Death Discov. 2026 Mar 7;12:143. doi: 10.1038/s41420-026-03003-3 (PMC13039842; doi:10.1038/s41420-026-03003-3)

*Blots were cuts to allow immunoblotting with
different antibodis without reblotting the whole memebrane

Fig. 2

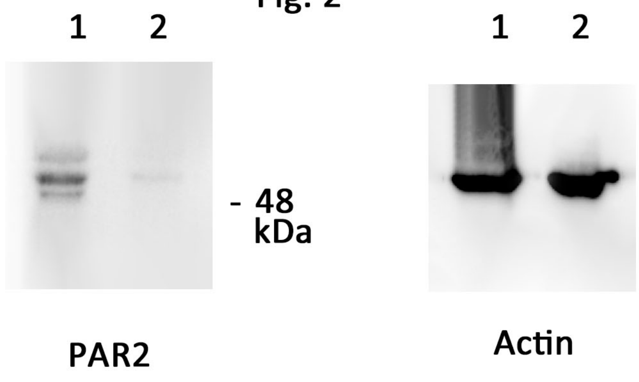

Fig. 3

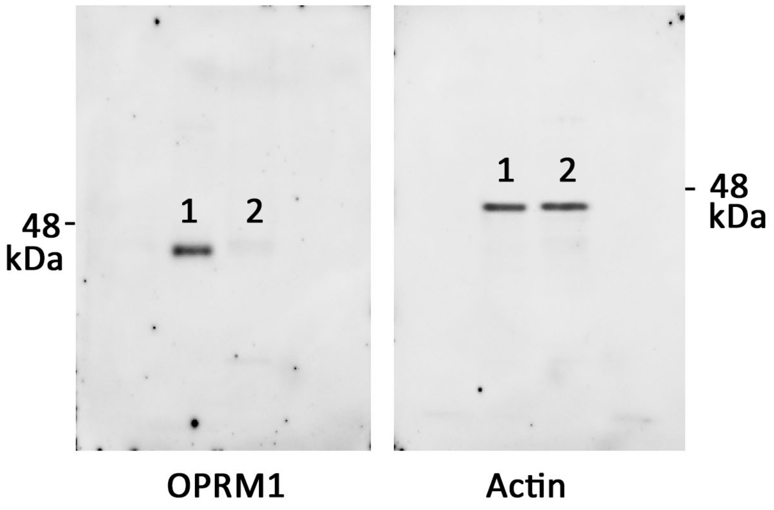

***Blots were cuts to allow immunoblotting with different antibodis without reblotting the whole memebrane**

Fig. 8A

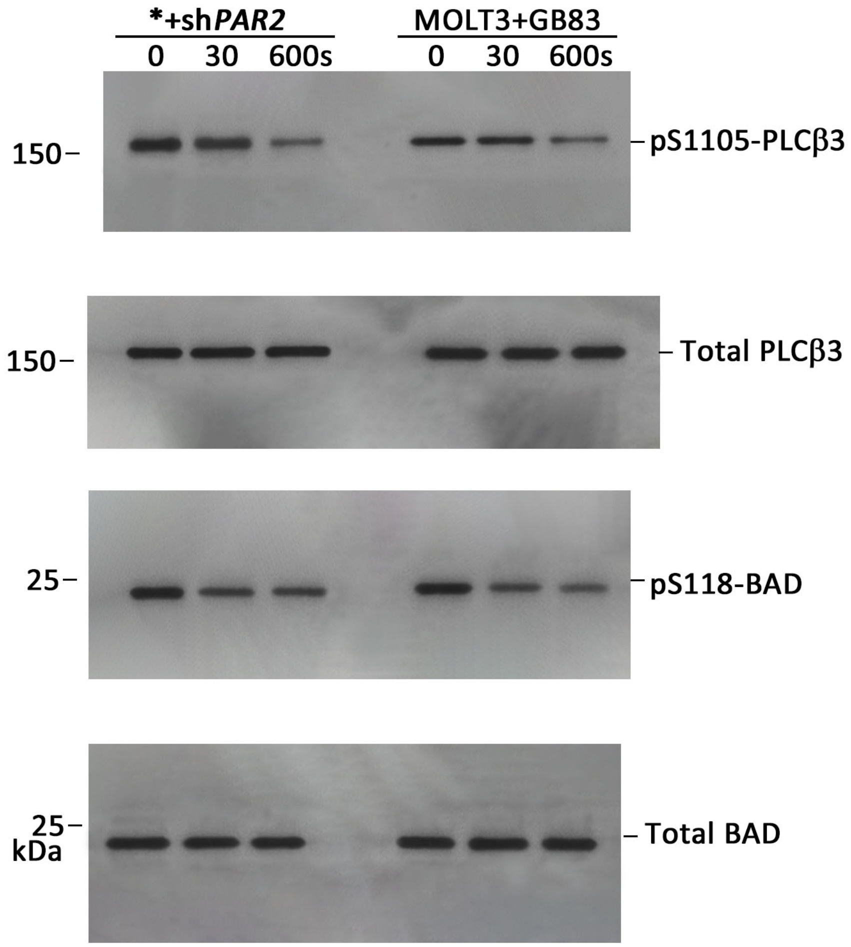

*Blots were cuts to allow immunoblotting with different antibodis without reblotting the whole memebrane

Fig. 8B

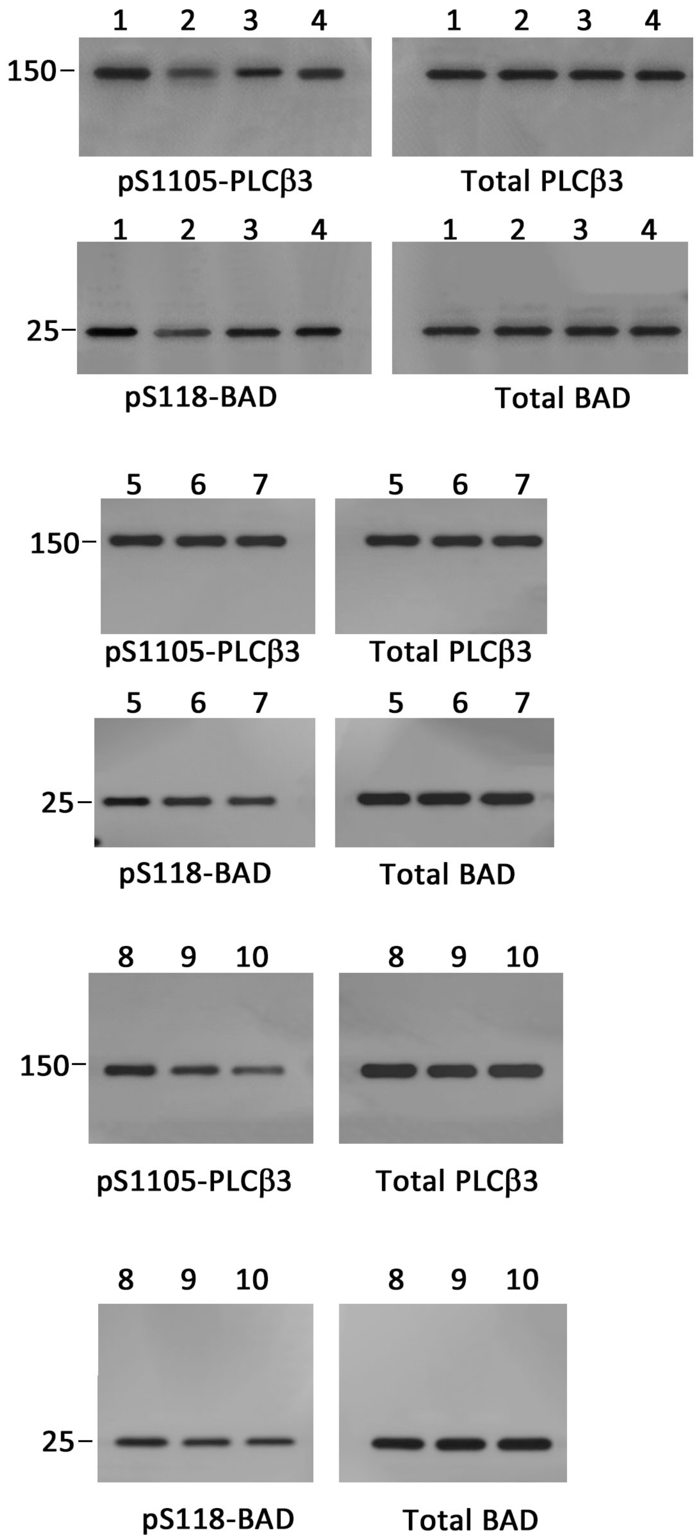

*Blots were cuts to allow immunoblotting with
different antibodis without reblotting the whole memebrane

Fig. 8C

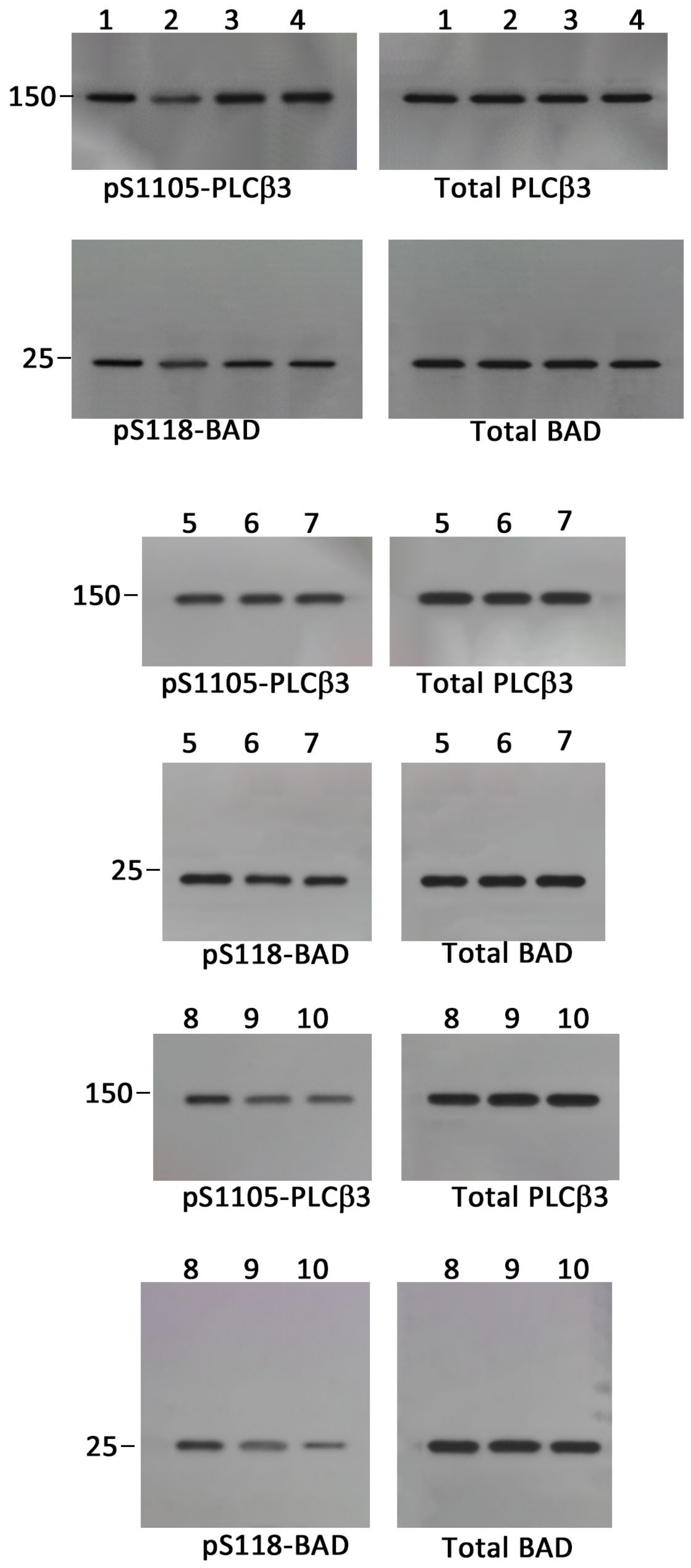

Supplement: Supplementary file 7 — Uncropped figures [file 41420_2026_3003_MOESM7_ESM.pdf]
